# Organisational models supported by technology for the management of diabetic disease and its complications in a diabetic clinic setting: study protocol for a randomised controlled trial targeting type 2 diabetes individuals with non-ideal glycaemic values (Telemechron study)

**DOI:** 10.1186/s13063-023-07515-6

**Published:** 2023-08-10

**Authors:** Alexia Giovanazzi, Lorenzo Gios, Marina Mastellaro, Maria Adalgisa Gentilini, Francesca Valent, Sara Condini, Giorgia Bincoletto, Alessandro Bacchiega, Andrea Zorzi, Giulia Malfatti, Francesca Perini, Claudio Eccher, Michele Marchesoni, Marlene Dall’Alda, Massimo Orrasch, Diego Conforti, Sandro Inchiostro

**Affiliations:** 1 Azienda Provinciale Per I Servizi Sanitari, Provincia Autonoma Di Trento, Trento, Italy; 2 TrentinoSalute4.0, Competence Center for Digital Health, Trento, Italy; 3 Servizio di Igiene e Sanità Pubblica, Dipartimento di Prevenzione, Azienda sanitaria universitaria Friuli Centrale, Gemona del Friuli, Italy; 4 Facoltà Di Giurisprudenza, Università Degli Studi Di Trento, Trento, Italy; 5 Fondazione Bruno Kessler, Trento, Italy; 6 Provincia Autonoma Di Trento, Trento, Italy

**Keywords:** Type 2 diabetes mellitus, Technology, Telemedicine, mHealth, Mobile application, Smartphone

## Abstract

**Introduction:**

Type 2 diabetes mellitus (T2DM) is a non-communicable disease representing one of the most serious public health challenges of the twenty-first century. Its incidence continues to rise in both developed and developing countries, causing the death of 1.5 million people every year. The use of technology (e.g. smartphone application—App) in the health field has progressively increased as it has been proved to be effective in helping individuals manage their long-term diseases. Therefore, it has the potential to reduce the use of health service and its related costs. The objective of this study is to evaluate the impact of using a digital platform called “TreC Diabete” embedded into a novel organisational asset targeting poorly controlled T2DM individuals in the Autonomous Province of Trento (PAT), Italy.

**Methods:**

This trial was designed as a multi-centre, open-label, randomised, superiority study with two parallel groups and a 1:1 allocation ratio. Individuals regularly attending outpatient diabetes clinics, providing informed consent, are randomised to be prescribed TreC Diabete platform as part of their personalised care plan. Healthcare staff members will remotely assess the data shared by the participants through the App by using a dedicated online medical dashboard. The primary end-point is the evaluation of the Hb1Ac level at 12-month post-randomisation. Data will be analysed on an intention-to-treat (ITT) basis.

**Discussion:**

This trial is the first conducted in the PAT area for the use of an App specifically designed for individuals with poorly controlled T2DM. If the effects of introducing this specific App within a new organisational asset are positive, the digital platform will represent a possible way for people diagnosed with T2DM to better manage their health in the future. Results will be disseminated through conferences and peer-reviewed journals once the study is completed.

**Trial registration:**

ClinicalTrials.gov NCT05629221. Registered on November 29, 2022, prior start of inclusion.

**Supplementary Information:**

The online version contains supplementary material available at 10.1186/s13063-023-07515-6.

## Administrative information

**Table:** 

**Title {1}**	Organisational models supported by technology for the management of diabetic disease and its complications in a diabetic clinic setting. Study protocol for a randomised controlled trial targeting type 2 diabetes individuals with non-ideal glycemic values (Telemechron study)
**Trial registration {2a and 2b}**	ClinicalTrials.gov NCT05629221 [Registered on November 29, 2022 prior start of inclusion]
**Protocol version {3}**	Version 2 of 27–09-2022 First issue date: 11 May 2022 (8 August 2022) Approved by Ethics Committee: 30 May 2022 (Amendment: 27 September 2022) (Ethics Committee opinions are available as Additional File 1) Primary reason for amendment: The trial initially aimed to recruit T2DM individuals with non-ideal glycemic values, also affected by post-infarction cardiomyopathy and/or heart failure. It was soon realised that the criteria of being both diabetic and with a heart condition would have compromised the recruitment process and no targeted sample size would have been reached in time. Therefore, the following two inclusion criteria were removed: (1) having a diagnosis of ischemic heart disease identified by a previous documented acute ischemic event requiring an hospital access or a diagnosis of ischemic heart diseases following a positive test for inducible ischemia, or (2) having a diagnosis of heart failure with a New York Heart Association (NYHA) Classification of Heart Failure I or II or III (reduced or preserved ejection fraction) [1] defined on the basis of a previous hospitalisation for heart failure, or defined by an ejection fraction below 50% and NYHA I or II or III (absence of dyspnoea at rest) Before the first version of the protocol was approved, Ethical Committee made the following requests: i) improved description of the intervention; ii) clarification related to the Regulation (EU) 2017/745 of the European Parliament and of the Council of 5 April 2017 on medical devices, that is, clarifying that TreC Diabete is not a medical device [2]; iii) amendment to the informed consent form to further simplify the wording (available in English upon request)
**Funding {4}**	This trial is funded by the Department of Health of the Italian National Health System in the context of the “*Ricerca finalizzata (Bando della ricerca finalizzata year 2018)*—National Research (Bandi/Call 2018)*”.* The TreC Diabete platform was designed and implemented by the Bruno Kessler Foundation (FBK), Italy. The platform is formally distributed by the Azienda Provinciale per i Servizi Sanitari (APSS). This is described in greater details in Additional File 2
**Authors detail {5a}**	In alphabetical order: Alessandro Bacchiega: Fondazione Bruno Kessler, Trento, Italy Giorgia Bincoletto: Facoltà di Giurisprudenza, Università degli Studi di Trento, Trento, Italy Sara Condini: Azienda Provinciale per i Servizi Sanitari, Trento, Italy Diego Conforti: Provincia autonoma di Trento, Trento, Italy Marlene Dall’Alda: Azienda Provinciale per i Servizi Sanitari, Trento, Italy Claudio Eccher: Fondazione Bruno Kessler, Trento, Italy Maria Adalgisa Gentilini: Azienda Provinciale per i Servizi Sanitari, Trento, Italy Lorenzo Gios: TrentinoSalute 4.0, Competence Center for Digital Health, Trento, Italy Alexia Giovanazzi: Azienda Provinciale per i Servizi Sanitari, Trento, Italy Sandro Inchiostro: Azienda Provinciale per i Servizi Sanitari, Trento, Italy Giulia Malfatti: TrentinoSalute 4.0, Competence Center for Digital Health, Trento, Italy Michele Marchesoni: Fondazione Bruno Kessler, Trento, Italy Marina Mastellaro: Azienda Provinciale per i Servizi Sanitari, Trento, Italy Massimo Orrasch: Azienda Provinciale per i Servizi Sanitari, Trento, Italy Francesca Perini: TrentinoSalute 4.0, Competence Center for Digital Health, Trento, Italy Francesca Valent: Azienda Provinciale per i Servizi Sanitari, Trento, Italy Andrea Zorzi: Fondazione Bruno Kessler, Trento, Italy
**Name and contact information for the trial sponsor {5b}**	Azienda Provinciale per i Servizi Sanitari, Provincia Autonoma di Trento, Italy Principal Investigator: Sandro Inchiostro Via Alcide Degasperi 79 – 38,123 Trento (Italy) Email: urp@apss.tn.it or sandro.inchiostro@apss.tn.it Telephone number: + 39 0461904111
**Role of sponsor {5c}**	The project "Telemedicine for the home management of patients with chronic diseases and comorbidities: analysis of current models and design of innovative strategies to improve the quality of care and optimise the use of resources: TELEMECHRON study" was presented to the Ministry of Health by a consortium of interregional entities composed of: Provincial Authority for Health Services of Trento; USL Toscana Nord Ovest (coordinator); Scientific Clinical Institutes Maugeri IRCCS; *Istituto Superiore di Sanità* (National Institute of Health). *Ministero della Salute* (Italian Ministry of Health) grant: NET-2018–12,367,206

## Introduction

### Background and rationale {6a}

Diabetes is a non-communicable disease characterised by an elevated level of blood glucose. About 422 million people have diabetes worldwide [3]; with 1.5 million deaths every year, diabetes is the fifth cause of death and represents one of the most serious public health challenges of the twenty-first century [3]. The most common type of diabetes, type 2 diabetes mellitus (T2DM), accounts for 90% of all diabetes cases. It is often described as a lifestyle disease because of its association with modifiable risk factors, such as overweight and physical inactivity. According to the World Health Organization (WHO), the incidence of T2DM is expected to rise in both developed and developing countries, leading to an increase in the economic loss for the people affected by the diseases, the society and the health systems [3]. Considering that diabetes is one of the main causes of severe long-term conditions, such as cardiovascular events, macro- and micro-vascular complications, it is urgent to tackle it by either preventing its onset or delaying its progression [4].

The management of diabetes is a sensitive issue, particularly when considering the (perceived) quality, acceptability and easiness of the service delivery, both from a patient and healthcare staff’s perspective [5]. WHO has advocated the use of mobile health (mHealth) as a potentially effective methodology to support patients in the management of their health [6]. MHealth is defined as a “medical and public health practice supported by mobile devices, such as mobile phones” for collecting, managing and sharing health-related data between patients—clinicians and researchers [6]. Therefore, mHealth constitutes a practice that can be effectively adopted for chronic diseases such as diabetes.

As part of the swift and disruptive development and use of technology in the context of public health and health care delivery that has characterised the last decades, there have been an increasing number of mHealth tools piloted in clinical contexts and targeting patients with diabetes [7]. A relevant number of studies tried to prove how communication technologies can increase self-management [8], as well as an effective improvement of clinical outcomes and better care costs [7, 9–11]. Recent systematic reviews and meta-analysis corroborate the effectiveness of mHealth intervention in improving clinical outcomes when compared to usual care in people with diabetes. The vast majority of these studies considered the improvement of the glycosylated haemoglobin (HbA1c) levels as their main outcome. The systematic reviews and meta-analysis of Martos-Cabrera and colleagues [12], Wu and colleagues [13] and Bonoto and colleagues [14], including both type 1 and type 2 diabetes, showed that the use of mobile apps could yield to a significant reduction of HbA1c in comparison to usual care. Authors discussed the importance of further investigating the use of mHealth since current studies regarding mHealth are very heterogeneous by population and mHealth interventions and likely to focus on short-term effectiveness only (< 12 months). The need of conducting more studies on mHealth intervention is supported by other studies results not able to find significant improvements in clinical outcomes, as reported in the systematic review and meta-analysis of Hyun and colleagues [15]. In this systematic review and meta-analysis considering T2DM adult participants and including 14 randomised controlled trials (RCTs), the superiority of using a mobile application (App) or an App with e-coaching on HbA1c levels could only be observed on a limited number of subjects and for a short-term follow-up time [15].

In order to respond to the WHO emphasis on the importance of investing in strategies and public health programmes to prevent and manage diseases through the use of mHealth [16], the Italian Ministry of Health has made funds available to conduct studies focusing on the application of technology in the management of chronic diseases.

This study protocol focuses specifically on one of the Work Packages (WP), i.e. WP4, of a wider project called “Telemedicine for home-based management of patients with chronic diseases and comorbidities: analysis of current models and design of innovative strategies to improve quality of care and optimise resource utilisation: TELEMECHRON study”. WP4 has been approved to be implemented also in the Autonomous Province of Trento (PAT), where, with a slightly lower prevalence of diabetes than in the Italian and global population, currently there are 28.000 cases of people diagnosed with diabetes (5.0% of PAT total population) [17, 18]. The aim of this specific WP is to comprehensively assess an innovative healthcare management system targeting patients with T2DM in non-ideal glycaemic control status. The system is enabled by the use of a mHealth platform called “TreC Diabete”, including an App for participants/patients and a web-based dashboard for health care staff.

### Objectives {7}

#### Research hypothesis

Participants accessing a highly integrated mHealth system can achieve an improved disease control with equal (or even lower) health service utilisation and greater satisfaction (from both patient and healthcare staff perspectives) compared to those not accessing it. The platform is also expected to act as a trigger for a closer interaction among healthcare professionals, such as specialists from other units and to simplify connections between healthcare staff and patient/caregivers.

#### Primary objective

The primary objective of this randomised two parallel groups study is to assess the impact of a novel organisational asset enabled by the use of the TreC Diabete platform on the level of HbA1c within a 12-month follow-up period.

#### Secondary objectives

Secondary objectives include the impact of the mHealth platform use in terms of control of blood pressure and cholesterol, modification of body weight, amount of physical activity, number of hospitalisations, adherence level to prescriptions, improvements of quality of life, satisfaction and usability of the application at different follow-up times within and between groups.

### Trial design {8}

The Telemechron study was designed as a multi-centre, open-label, randomised, superiority study with two parallel groups and a 1:1 allocation ratio. This study design was chosen over others because RCTs are considered the best type of study design to evaluate the effectiveness of health interventions [19].

## Methods: participants, interventions and outcomes

### Study setting {9}

The participants are recruited in the diabetic outpatient clinics of the Hospitals of Rovereto and Trento, which are the most relevant diabetic clinics of PAT for diabetic individuals, with an average of 30 visits per day at Rovereto, and 40 visits per day at Trento. In the Province, the prevalence of T2DM is estimated to be 5.0%. This is slightly lower prevalence than the one estimated nationally (i.e. 6.2%) [17].

### Eligibility criteria {10}

Participants must meet at randomisation all the inclusion criteria and none of the exclusion criteria listed in Table 1.

**Table 1:** Inclusion and exclusion criteria

Inclusion criteria	Exclusion criteria
• Being diagnosed with T2DM; • Aged ≥ 18 and ≤ 85 years old; • Having an HbA1c level > 7% (53 mmol/mol) and < 12% (108 mmol/mol); • Being able to walk without walking aids; • Having provided written informed consent; • Having a smartphone or a tablet and being able to download the App that will be used to insert the required data	• Having a BMI < 18 kg/m^2^ and > 45 kg/m^2^; • Having a sBP < 100 or > 200 mmHg and/or dBP < 50 or > 120 mmHg; • Being diagnosed with a stage 5 CKD^a^ • Being diagnosed with a NYHA stage IV; • Poor collaboration (i.e. unwilling to modify the actual plan of control and therapy, even if not ideal) for which adherence to the study is unlikely • Having no possibility of using a smartphone or a tablet • Having medical conditions affecting the study participation (i.e. life expectancy < 1 year)

*Abbreviations*: *BMI* body mass index, *CKD* chronic kidney disease, *dBP* diastolic BP, *HbA1c* glycated haemoglobin type A1c, *NYHA* New York Heart Association, *T2DM* type 2 diabetes mellitus, *sBP* systolic BP

^a^based on the glomerular filtration rate according to the CKD-Epidemiology Collaboration equation < 15 ml/min/m^2^ accounting for individual body surface [20]

### Who will take informed consent? {26a}

Potentially eligible participants are identified during diabetes clinic appointments, either face-to-face or via a remote appointment (phone call). Clinicians of the diabetes clinics will provide standardised and key information regarding the study. If patients are interested, they will receive the information sheet to read (either face-to-face or by email). The information sheet and the consent form for the participants are originally written in Italian. At this stage, patients will be contacted by a member of the research team (registered nurse) who will also ensure that the information sheet is fully read and understood. During another dedicated face-to-face appointment, the patient who volunteers to participate in the study will provide written informed consent, provided that all questions regarding his or her involvement in the study are answered. Clinicians are responsible to sign the informed consent form with the patient during this visit.

### Additional consent provisions for collection and use of participant data and biological specimens {26b}

In addition to the informed consents for the study, the researchers collect the informed and specific consents for the personal data processing activities. In fact, the processing of personal data of this research should comply with the General Data Protection Regulation (Regulation 2016/679 or “GDPR”) and the Italian Data Protection Code (*Decreto Legislativo*—D.Lgs 101/2018) [21, 22]. The privacy policy has been elaborated under Articles 12 and 13 of the GDPR. The legal grounds of the processing are both the explicit consent of the data subjects (as regards data concerning health) and the performance of tasks carried out in the public interest by the data controller.

By signing the informed consent, participants will allow the appointed researchers to access their medical records’, including blood test results at follow-up. Blood tests are carried out by qualified nurses who are not part of the research team but are still part of the APSS organisation. Blood tests performed within the hospital organisation do not require additional written consent.

## Interventions

### Intervention description {11a}

After being approached for face-to-face screening and enrolment, eligible individuals are randomised on a 1:1 allocation ratio to the intervention or the control group.

Those allocated to the intervention group are prescribed with the TreC Diabete App. This is one of several Apps created within the so-called TreC platform, which enables citizens from PAT to access, manage and share information about their health and wellbeing in the context of telemedicine [23–26]. TreC is an acronym for *Cartella Clinica del Cittadino* (Citizens’ Clinical Records), in which the citizen/patient plays the role of the focal manager and owner of his/her own health data. The technological platform has been preliminarily used for other studies targeting patients with diabetes, specifically pregnant women [27].

Participants will download the App from Google Play or Apple Store (Android or iOS system, respectively), accept the online information form, enter their tax code (*Codice Fiscale*) and the health card number, create a PIN code and then confirm it to complete the procedure. At this point, the participant must wait for the clinician to proceed with the activation of the App; otherwise, it will not be possible to access the functions. Participants are enabled to activate the App as soon as the clinician provides the unique activation code. Participants can then set a personal password, required to access the App.

Clinicians will then prescribe the App for a 12-month period through the online medical dashboard. The dashboard allows the visualisation of the information shared by the participant. Moreover, it enables healthcare staff to interact directly through video-chat and chat with the participant. A menu allows clinicians to set up a specific and personalised plan for each participant: they are asked to indicate which parameter (e.g. BP) should be registered in the App and to activate reminders at set intervals. During the 12-month follow-up, clinicians will regularly check the information entered by the participant (minimum frequency of 45 days). Clinicians can provide advice based on the information shared by the participant (e.g. inviting the participant to book an appointment with their general practitioner—GP). Participants will receive the training for the use of the App as soon as they are allocated to the intervention group. A member of the research team (registered nurse) will show the participant how the App works, highlighting its different features. The appointed person will specifically show how to enter the PIN when asked, insert different types of data in the App, write in the chat, and fill in the questionnaire. Also, the registered nurse could be contacted by phone in case of problems managing the app. The app’s chat was another way to contact researchers and IT personnel to solve management problems. Paper-based training material can be provided to the participant upon request.

Table 2 depicts the App features (for the prescriber and the participant perspectives) and provides an explanation of their use.

**Table 2:** TreC Diabete App features and related description

Level	Feature	Description
Dashboard (prescriber)	Activation of credentials for the participant	The feature allows to activate the App for a specific participant and is performed by the clinicians via dashboard
Dashboard (prescriber)	Setup of the data each participant will be required to enter into the App	The feature allows the clinician to decide which data the participant will be asked to enter by the App. It allows the storage of the data and the sharing of information
Dashboard (prescriber)	Display of data	The feature allows the user to view the data as entered by the participant. It allows the sharing of the information, which cannot be altered or modified
Dashboard (prescriber)	Activation and use of a chat between clinicians/healthcare staff and participants	The feature allows the user to activate a chat for remote contact and communication between clinicians and participants. Different types of information could be shared through this channel (including files and pictures)
Dashboard (prescriber)	Personalised activation of tasks based on the diabetes profile	The feature allows the clinician to assign tasks and related reminders regarding the insertion of certain parameters, such as weight, blood pressure, glycaemia, with a defined frequency, time of the day of execution and related deadlines. The function allows the memorisation and the communication of information
Dashboard (prescriber)	Setup of reminders related to drugs	The feature allows the clinicians to set reminders regarding specific drugs and healthy lifestyle behaviours. It is considered a function that allows the communication of information
Dashboard (prescriber)	Set up of a blood glucose profile	The feature allows the clinician to insert the participant’s blood glucose profile for informational purposes only
Dashboard (prescriber)	Set up of ordinary reports	The feature allows the doctor to receive regular reports produced by the App and based on the data inserted by the participant at specific intervals. The data are shown as aggregated and cannot be modified
App (participant)	Chat between clinicians and participant	The feature allows the participant to send messages to the clinician for remote contact and communication
App (participant)	Activation of the diary of personal observations	The feature allows the participant to enter and review the parameters inserted for the activated tasks. Reminders may be activated (without push notifications). Participants can enter and insert other unassigned parameters. The insertion is done directly by the participant. The diary is not directly visible to the clinician through the dashboard with the exception of the prescribed parameters
App (participant)	Activation of the drugs’ diary	The feature allows the participant to manually record the intake of drugs (either prescribed or not by the clinician)
App (participant)	Set up of reminders	The feature allows the participant to receive notifications (push) concerning telemedicine appointments

Participants in the control group are asked to record their health-related data such as body weight, BP, glycaemic levels, results of laboratory tests and diagnostic tests as usual (e.g. on a paper diary). Other information collected by the control group participants during the follow-up period can be shared, as routinely done, with their GP.

For the App functions described above and according to the Medical Device Regulation (MDR) (EU) 2017/745 requirements [2], the TreC Diabete App cannot be considered a medical device. The App does not perform operations on clinical data; it is used as data archive and for communication and simple research purposes. It does not provide direct recommendations to participants based on algorithms; it does not make changes or alterations to the collected data and it does not transfer data with implantable devices eventually belonging to the participants. Moreover, the App does not provide direct recommendation to the clinicians for diagnosis and treatment; it cannot be used to handle emergency situations.

Table 3 depicts the list of the therapeutic goals that people with T2DM should reach set by the current national and international guidelines [28–31].

**Table 3:** List of therapeutic goals as set by the current national and international guidelines [28–31]

Type of target	Values
Glycaemic target	HbA1c < 53 mmol/mol
BP target	sBP < 130 mmHg if < 65 years old sBP < 140 mmHg if ≥ 65 years old dBP ≥ 70 mmHg and < 80 mmHg
Lipid target	LDL cholesterol < 70 mg/dl non-HDL cholesterol < 100 mg/dl (if triglycerides ≥ 200 mg/dl)

*Abbreviations*: *dBP* Diastolic blood pressure, *HDL* High-density lipoproteins, *LDL* Low-density lipoproteins, *sBP* Systolic blood pressure

### Explanation for the choice of comparators {6b}

Both the intervention and control group will receive the best care for T2DM, in line with the integrated preventive and diagnostic care pathway (called “*Percorso Preventivo Diagnostico Terapeutico Assistenziale*—PPDTA” in Italian) which is available in Italian upon request to the authors of this manuscript. An appointment with the outpatient diabetes clinic staff is scheduled at 6 and 12 months from the first visit (taking place within 30 days of the study entry), either through telemedicine (remote visit) or face-to-face for all participants.

### Criteria for discontinuing or modifying allocated interventions {11b}

Participants can leave the study at any time and for any reason if they wish to do so.

### Strategies to improve adherence to interventions {11c}

Adherence to the study will be monitored by the research team and clinicians. Study participants allocated to the intervention group will receive set up reminders through the App regarding the tasks that need to be done (e.g. measure and record the blood sugar level). Adherence to the study will be monitored by the research team and clinicians, as usage and interaction by the participant with the App can be checked through the dashboard. Scheduled visits at the diabetes centres can also be an opportunity to discuss possible difficulties in using the App and to remind participants why it is important to regularly record their data.

### Relevant concomitant care permitted or prohibited during the trial {11d}

Concomitant care is permitted in line with the local standard practice. In case of relevant care actions (e.g. emergency), data are recorded in line with the standard procedures as part of the patients’ record.

### Provisions for post-trial care {30}

Post-trial care is delivered in line with the local standard practice.

### Outcomes {12}

#### Primary outcome measure

- Difference in the participants’ HbA1c level between the two arms at 12 months. Blood tests, including HbA1c level, are carried out by qualified nurses and processed by the local analysis laboratory. Blood tests results are automatically registered on the Hospital Information System (HIS) and reviewed by clinicians and research staff involved in the study.

#### Secondary outcome measures

Secondary outcome measures are collected at different time point (for specific times of data collection refer to Table 4).

- Proportion of participants with Hb1Ac < 53 mmol/mol within the two arms;
- Difference in HbA1c levels between the two arms other than at 12 months;
- Reporting frequency and number of new hypoglycaemic episodes within the two arms;
- Proportion of participants reaching the targeted lipid profile (LDL level < 70 mg/dl and non-HLD cholesterol level < 100 mg/dl) and BP levels (sBP < 130 mmHg or > 140 if ≥ 65 years old);
- Effect on weight, lipid profile and BP levels for the App users;
- Effect on physical activity performed during the last 7 days prior filling in the questionnaire, for both arm participants;
- Number of hospitalisation events within the two arms;
- Number of measurements and level of physical activity with the prescribed activities (body weight monitoring, BP measurements, physical activity) within the two arms;
- Changes in quality of life (QoL) scores for participants of both arms;
- Satisfaction and usability score of the App in the intervention arm;

**Table 4:** Time schedule of enrolment, interventions and assessments during the study period, adapted from the example template provided by the SPIRIT Group [28]

	Enrolment		Allocation		Post-allocation			Close-out	Personnel and participant involvement	Method of data collection
Timepoint	14 to 21 days prior T0	7 to 14 days prior T0	0 months	0 months (T0)	3 months (T1) (± 30 days)	6 months (T2) (± 30 days)	9 months (T3) (± 30 days)	12 months (T4) (± 30 days)		
ENROLLMENT										
** Informal study information**	x	x							Clinician	Scheduled appointment at clinic
** Eligibility screen**	x	x							Clinician or PI	Scheduled appointment at clinic
** Informed consent signed**		x							Clinician or PI and participant	Face-to-face meeting
** Allocation**			x						Research team	Face-to-face meeting
** Blood tests prescription**		x							Clinician	Face-to-face meeting
** Provide 3 copies of IPAQ questionnaire**		x							Research team	Face-to-face meeting
INTERVENTION										
** App education and instruction manual**				x					Research team	Face to face meeting
** Inform GP of participation**				x					Clinician	Through email
** Help and support with the App use**				x	x	x	x	x	Research team	Phone- email
** App (intervention) monitoring**				x	x	x	x	x	Clinician or research team	Medical dashboard
** Medical intervention via App – data recording**				x	x	x	x	x	Clinician and participant	Medical dashboard
ASSESSMENTS: clinical and biohumoral variables and clinical indicators										
** Body weight and number of checks (previous month)**		x			x	x	x	x	Research team	Face to face meeting, medical dashboard /App or other recording tools
** Vital signs and number of checks (previous month)**		x			x	x	x	x	Research team	Face to face meeting, medical dashboard/App or other recording tools
** Demographic characteristics and clinical variables**^**a**^		x							Clinician and research team	Face to face meeting, HIS
** Hb1Ac levels and number of checks**		x			x	x	x	x	Clinician, research team, laboratory personnel	HIS
** Other blood tests**^**b**^		x						x	Clinician, research team, laboratory personnel	HIS
** Questionnaires (SF-12/MMAS-4/DASI)**				x				x	Research team, participant	Face to face meeting
** IPAQ questionnaire**				x	x	x	x	x	Research team, participant	Face to face meeting and phone call
** SUS questionnaire (App group only)**								x	Research team, participant	Face-to-face meeting
** Number of nursing and medical (tele)consultation and chat interactions**					x	x	x	x	Research team	Medical dashboard/App and HIS
** Number of unscheduled specialists visits (macro- and micro-vascular complications), number of accesses to ED for cardiac, cardiovascular or diabetic problems**					x	x	x	x	Research team	HIS
** Number of therapeutic plan changes**					x	x	x	x	Research team	Medical dashboard/App and HIS
** Time spent for (tele)consultation**						x			Research team	Recording of time during visit
** Number of hospitalisation**						x	x	x	Research team	HIS
** Number of hypoglycaemic episodes**					x	x	x	x	Research team, participants	Medical dashboard/App and/or download of data from glucometer

*Abbreviations*: *DASI* Duke Activity Status Index, *ED* emergency department, *HbA1c* Glycosylated Haemoglobin Type A1C, *HIS* Hospital Information System, *IPAQ* International Physical Activity Questionnaire, *MMSA-4* Morisky Medication Adherence Scale, *SF-12* Short Form-12, *SUS* System Usability Scale, *PI* Principal Investigator

^a^demographic characteristics: age, sex, citizenship, marital status, school level; clinical variables: diabetes diagnosis year, smoking habit, weight, height, body mass index, systolic and diastolic blood pressure, heart rate, last emergency hospital admission for cardiologic condition, retinopathy, neuropathy, acute myocardial infarction, heart failure, ischemic heart disease, previous cerebrovascular events, obliterative arterial disease in the lower limbs, chronic kidney disease, dementia, chronic obstructive pulmonary disease, rheumatologic disease, peptic ulcer, liver disease, diabetic and cardiovascular drug therapies ^b^fasting glycaemia, total cholesterol, HDL cholesterol, non-HDL cholesterol, LDL cholesterol, creatinine, eGFR (CKD-EPI), ratio albumin/creatinine on morning urine sample, triglycerides

#### Process indicators

To assess the organisational implications of this novel approach the following process indicators will be collected:

- Number of telemedicine visits between participants and nurses and chat interactions
- Number of telemedicine visits between participants and clinicians and chat interactions
- Number of (tele)specialist consultations (e.g. cardiology visit)
- Number of changes in the therapeutic plan (e.g. drug prescription and dosage)
- Amount of time spent for telemedicine visits (calculated on the basis of the average time spent to deliver eight consecutive nursing visits and eight consecutive medical visits, as delivered at 6 months).

### Participant timeline {13}

Participants will be followed for 12 months (± 30 days)—T4—starting from the allocation time (T0). Post-allocation evaluations are set to be at 3, 6 and 9 months (± 30 days)—(T1, T2 and T3, respectively). Table 4 depicts the time schedules of enrolment, interventions and assessments at different time points [32].

### Sample size {14}

**Fig. 1:**
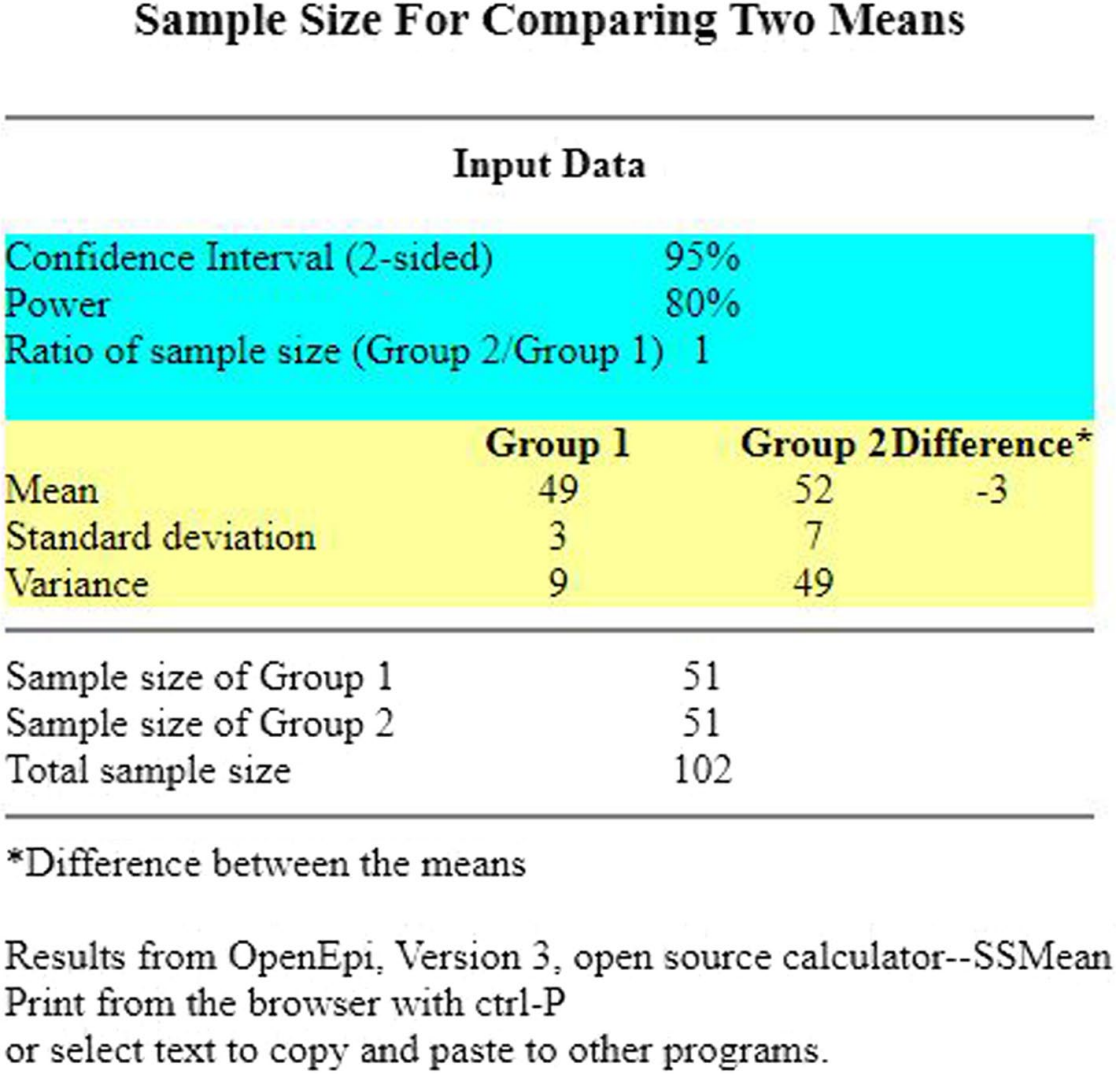
Sample size calculation—output from Open Epi

The sample size was calculated on the basis of the primary hypothesis (Hb1Ac levels are likely to decrease more in the subjects of the intervention group than for the subjects of the control group at 12 months). A lower variability around the HbA1c mean level is also expected for the subjects in the intervention group when compared to the HbA1c mean level for the subjects in the control group. It was assumed that at 12 months, the mean Hb1Ac level will be 49 (SD ± 3) mmol/mol in the intervention group and 52 (SD ± 7) mmol/mol in the control group. In order to achieve a power of 80% and to keep the significance level at 5%, the sample size was calculated to be 102 (51 in each group) by using Open Epi (a freely accessible software, https://www.openepi.com/Menu/OE_Menu.htm) as depicted in Fig. 1 below. Assuming 20% of drop-out, the required sample was calculated as 120.

### Recruitment {15}

Patients regularly attending diabetes clinics in PAT are invited to participate if meeting the eligibility criteria. In order to identify the eligible participants, clinicians will use the list of patients with a scheduled appointment at the clinics (either face-to-face or online) and then check their medical history through the HIS database. Recruitment will stop when the targeted sample size is reached. Enquires (by phone or by email) from the potential participants are answered by the staff of the clinics during business hours.

## Assignment of interventions: allocation and blinding

### Sequence generation {16a}

Stratification is applied by centre (Rovereto and Trento). Participants are randomised (block randomisation with block size of 10) to either the intervention or control group on a 1:1 basis.

### Concealment mechanism {16b}

Treatment assignment information is enclosed in opaque envelopes, which are unsealed once the participant provides the written informed consent and the baseline information is collected.

### Implementation {16c}

All patients who give consent for participation and who fulfil the inclusion criteria will be randomised.

The random allocation sequence was generated by one statistician from the Clinical and Evaluation Epidemiology Unit of the Local Health Trust (APSS of the PAT), not involved with the recruitment and enrolment of the participants. The statistician used a SAS macro applying blocks following stratification by centre of recruitment. In every centre, closed opaque envelopes with printed randomisation numbers on it are available. For every randomisation number, the corresponding code for the parallel groups of the randomisation list will be found inside the envelopes.

A dedicated member of the research team will open the envelope and will find the allocated group for the participant. Then, the same member of the research team will give the information about group allocation to the patient.

### Who will be blinded {17a}

Given the nature of the intervention, which is the active use of the Smartphone App, it will not be possible to guarantee the blindness of participants, healthcare staff and the person responsible for data collection. Vital signs, body weight and height are recorded before randomisation by a trained nurse across sites, using standardised procedures and instruments. The blood tests’ analysis will be carried out within the Trusts according to standardised procedures. Laboratory personnel are not involved in the study, thus they are not aware of the participants’ allocation. Blindness is guaranteed during data analysis, as data analysis will be performed from assessors that are not directly involved in the study. Filled-in questionnaires will only report the unique randomisation number.

### Procedure for unblinding if needed {17b}

This is an open-label trial, in which only data analysts will not be aware of participant allocation. Therefore, unblinding of treatment allocation to the participants or the staff involved in the care of the participants will not be an applicable procedure for this specific trial.

## Data collection and management

### Plans for assessment and collection of outcomes {18a}

The large majority of the data (e.g. demographic data, clinical information) are collected as part of the standard routine practice. Other data are collected through interviews and questionnaires that are provided during different times of the study. Those assigned to the intervention group will be asked to provide information through the App (e-diary and Virtual Coach), whilst those assigned to the control group will register the data as per routine (e.g. paper diary). Please refer to Table 4 under the “Outcomes {12}” section for the information regarding the involvement of personnel—participants and the source of information used for data collection.

Below, a short description of the questionnaires used during this study is provided:

1. Quality of life questionnaire “SF-12”: composed of 12 items (obtained from 36 items of the original SF-36 questionnaire) and providing two measures related to two different aspects of health: physical health and mental health [33, 34].
2. Activity status evaluated through the “Duke Activity Status Index—DASI scale”, which measures the functional capacity perceived by the participant. It is a 12-item scale with a score that ranges from 0 (worst) to 58.2 (best). A difference of 2 or more is considered clinically significant [35, 36].
3. Daily physical activity level, evaluated using the “Physical Activity Questionnaire Daily – IPAQ” questionnaire, composed of 4 generic items with open-ended questions regarding last 7-day recall of physical activity [37];
4. Evaluation of medication adherence using the Morisky Medication Adherence Scale in the 4-item version (MMAS-4) [38]. The scale consists of 4 questions with a score of 0 and 1 for a “Yes” and “No” answer, respectively. The questions are summed to give a range of scores from 0 (high adherence) to 4 (low adherence). The questionnaire answers will be collected during the face-to-face meeting with the research team.
5. App user satisfaction using the System Usability Scale (SUS): The scale consists of 10 questions with a score ranging from 1 (strong disagreement) to 5 (strong agreement). Each score is then multiplied by 2.5, resulting in a total score ranging from 0 to 100. A 68 score is considered above average [39].

### Plans to promote participant retention and complete follow-up {18b}

Retention is critical to the success of our study, whose aim is to assess if the TreC Diabete platform will positively impact on clinical outcomes of T2DM subjects. Participants are invited to complete questionnaires and to undertake blood tests regardless of their level of engagement with the App. Moreover, participants will provide informed consent for the researcher team to access HIS database data, enabling them to collect information on clinical outcomes even if participants are not going to complete the study as planned. The regular visits planned at the Diabetic Clinics will allow nurses and clinicians to invite participants to use the App more frequently whenever the App is used in a suboptimal way. Lastly, we expect that frequent reminders will help the intervention group participants to engage with the App. No study within a trial (SWAT) was planned for our study.

### Data management {19}

#### Data forms and data entry

 Data are entered electronically in the Microsoft Access (version 2007) programme package. The file containing all the information is protected by a password and shared only within the research team. The HIS database, from which clinical outcomes data and other indicators are going to be collected, will only be accessed at the participating sites (Hospitals of Rovereto and Trento) to ensure data security. Each member from the staff has a personal password and can only access the HIS database under specific authorisations. Participant files will be maintained in storage for a period of 3 years after completion of the study.

#### Data transmission and editing

 Data integrity is guaranteed through different approaches, ranging from valid value assessment, range and consistency checks, when data entry is implemented, where the different pieces of information will be retrievable for viewing through the data entry system. Specific privileges are assigned to users in line with their user identification code and password.

#### Data discrepancy inquiries and reports to core coordinating centres

 A system to detect missing data or specific errors is implemented, and the identified Data Manager is in charge of cross/check the original forms and other sources—if needed—in case of inconsistency, and finally amending the form accordingly. Each inquiry received will be managed; otherwise, the queried item will not be available for closure.

#### Security and back-up of data

 Each coordinating centre will have possibility to access only to the data pertaining to the centre itself, whilst access’ passwords will be changed on a regular basis in line with internal procedures. A daily back-up of the primary database will be guaranteed, whilst back-ups of periodic data analysis files will be stored. Once the study is completed, the final database is available on the server of APSS. After 3 years, the data will be deleted or anonymised.

#### Study status reports

 Monthly reports with information on missing data will be produced for review and management purposes.

#### Description of hardware

 Personal computers connected with APSS server and intranet network, which can be accessed through a unique username and password.

### Confidentiality {27}

Information collected through the study is stored securely in locked cabinets at the study sites. Locked cabinets are located in rooms which are accessible only by healthcare and research staff members. Any form containing personal identifiers are located separately from the study records forms containing the subjects’ unique code number through pseudonymisation techniques. Databases used for data collection are secured with personal passwords and can only be accessed by the authorised research staff.

### Plans for collection, laboratory evaluation and storage of biological specimens for genetic or molecular analysis in this trial/future use {33}

Biological specimens will not be used for future search. Blood tests are carried by nurses of the Trust as outlined in the protocol. Once the results are reported on HIS, blood samples are destroyed.

## Statistical methods

### Analysis primary and secondary endpoints {20a}

The analyses of the primary and secondary endpoints will be performed based on the intention-to-treat and as per-protocol approaches. Continuous variables of the two groups will be presented as mean, SD and quartiles, whilst categorical variables will be presented as percentages.

The normality of the distributions is determined by using the normality test of Kolmogorov–Smirnov. To compare the differences between the two groups for continuous outcomes, Student’s *T* test will be used. If the assumption of normality of data distribution does not hold, Wilcoxon’s rank sum test will be used. For repeated measures of continuous variables for which there is an assumption of normality of data distribution, the one-way ANOVA test will be used. If the assumption of normality does not hold, the Friedman test will be used instead of the one-way ANOVA test.

Chi-squared test will be used for categorical outcomes. SAS 9.4 (SAS Institute Inc.) will be used to conduct analyses. For all tests, the significance level is set to 0.05 (2-sided). Professional statisticians (FV, MG) will conduct all analyses.

### Interim analyses {21b}

No interim analysis is planned. The study will stop in case the targeted sample size is not reached within the end of the funded initiative (Telemechron project is expected to end in late 2023) despite all efforts have been put in place to recruit participants. No harm is expected, and therefore, there are no other reasons why the study should be prematurely stopped.

### Methods for additional analyses (e.g. subgroup analyses) {20b}

No subgroup analyses were planned.

### Methods in analysis to handle protocol non-adherence and any statistical methods to handle missing data {20c}

The analysis will be conducted by “intention to treat” and “per protocol”. Given our expectation that very few patients will crossover or be lost to follow-up, these analyses should agree very closely.

Missing data will be managed according to the SAS version 9.3 MI procedure, which consists of the multiple imputation methods chosen by pattern of data missingness and type of imputed variable (SAS Institute Inc.)

### Plans to give access to the full protocol, participant-level data and statistical code {31c}

Data are not going to be available on an individual level, but only through aggregated form upon reasonable request.

## Oversight and monitoring

### Composition of the coordinating centre and trial steering committee {5d}

The steering group is composed by healthcare professionals and experts from the different institutions involved in the local initiative, namely APSS, PAT, Bruno Kessler Foundation (FBK) (i.e. research and IT innovation institute) and TrentinoSalute4.0 (i.e. Competence Center for Digital Health of the Province of Trento).

The technical committee in charge of coordinating the implementation of the study is composed by experts from APSS (clinical and organisational component, statistical component, data management and analysis) and FBK (IT component), with the support of TrentinoSalute4.0 (Competence Center for Digital Health of the Province of Trento). Patient and public groups were not involved for this specific trial; however, the platform under investigation is similar to those developed for other studies, for which both patients and health care providers had been involved [27].

### Composition of the data monitoring committee, its role and reporting structure {21a}

A data monitoring committee is not involved for this study.

### Adverse event reporting and harms {22}

Due to the nature of the intervention, no harm is expected. However, cyber security for both medical dashboard and the App needs to be guaranteed in order to protect personal data of the participants involved in the study. Healthcare staff members will receive appropriate training in the use of the technological platform. A username and a password will be provided to each healthcare staff member involved in the study, who will then be able to access the platform only by entering their username and personal password. A help desk is available to help and support the participant as well as the healthcare staff involved in the project. Efforts have been put in place and will be put in place to protect personal data in compliance with the EU GDPR 2016/679 and the subsequent national Italian laws, including the Data Protection Code (D.lgs. 196/2003) [21, 22]. The data controller and processor has implemented technical and organisational measures to safeguard data protection principles (Art. 5 GDPR) and guarantee the rights to the data subjects (Artt. 15–21 GDPR). Data collected will be pseudo-anonymised and pooled before being shared with third parties, as specified in the privacy policy that participants have to access and sign upon the start of the study. The data processor has been designated through a specific contract under Art. 28 of the GDPR and the data protection officer of both the data controller and processor will support the compliance with the legal requirements. Episodes of hospitalisation or complications due to T2DM itself (e.g. hypoglycaemic episode) will not be considered as an adverse event of the trial intervention or trial conduct.

### Frequency and plans for auditing trial conduct {23}

No auditing was planned for this study. The Ethical Committee will be informed on the progress and emerging issues throughout the whole study period.

### Plans for communicating important protocol amendments to relevant parties (e.g. trial participants, ethical committees) {25}

Any request for substantial amendments will be sent for evaluation to the Local Ethics Committee of the Healthcare Trust of PAT. The Ethics Committee will also be notified if there are any changes in the protocol that do not require substantial amendments. If amendments concern directly the participants, participants will be promptly informed about the changes.

### Dissemination plans {31a}

The results of the study, either positive or negative, are planned to be published in a peer-reviewed journal. They will also be disclosed through conferences.

## Discussion

This research project aims at assessing the impact of a telemedicine platform dedicated to DM patients as a self-management tool for supporting empowerment, self-measurement of parameters and adherence to care.

On the basis of previous studies, the TreC Diabete platform has proven to be an acceptable and viable tool to support new healthcare service delivery approaches enabled by new technologies: previous studies highlighted the use of TreC Diabete particularly among pregnant women, and the platform is currently part of the routine practice for this specific target considering the enrolment of approximately 100 women per year [23, 27]. This is done considering the complexity of the specific pathology with comorbidities and with respect to the treatment path with standard treatment. The use of a dedicated App for supporting patients’ empowerment, simplifying connections between healthcare staff and patient / caregiver, is expected to improve process, intermediate and final outcomes of the patients’ disease. Furthermore, the telemedicine platform is expected to represent a factor of satisfaction for patients, without a relevant impact in terms of workload for the healthcare staff. The platform is also expected to act as a trigger for a closer interaction among healthcare professionals. Interfacing with healthcare professionals asynchronously can promote patient compliance and allow healthcare professionals to arrive at the time of the visit with the anamnestic, clinical and laboratory elements ready. Potentially, this aspect could allow to reduce the timing of the visits whilst maintaining and even increasing the quality level of the diabetic visit. This aspect is particularly important in light of the challenge that has arisen in recent years and in future years of managing a complex, disabling and progressively growing disease, to be faced with resources that can be increased with difficulty in parallel with the spread of diabetic disease.

### Trial status

The study protocol was approved by the Local Ethics Committee for Clinical Trials of the Healthcare Trust (APSS) of PAT (Rep. Int. 9453–30/05/2022; project ID: A765 and Rep. Int. 17,489 -11/10/2022; project ID: ES463). The trial is currently ongoing and is planned to finish by September, 2024. Recruitment is planned to be completed by April 2023.

### Supplementary Information

**Additional file 1.** 

**Additional file 2.**

## Data Availability

The Principal Investigator will have full access to the data collected. Participants’ clinical data are accessible to the clinicians and other healthcare staff of the diabetic clinic via HIS provided that a reasonable justification is given. Data shared by the participant through the TreC Diabete App can be visualised by the treating clinicians at any time. Technical and IT maintenance will be guaranteed for the platform and App by technicians formally appointed to access the platform exclusively for maintenance reasons. However, technicians will only be able to visualise pseudo-anonymised data.

## Abbreviations

APSS: Azienda Provinciale per i Servizi Sanitari
CKD: Chronic kidney disease
dBP: Diastolic blood pressure
DASI: Duke Activity Status Index
D.Lgs: Decreto Legislativo
DM: Diabetes mellitus
FBK: Bruno Kessler Foundation
GDPR: General Data Protection Regulation
GP: General practitioner
HbA1c: Glycated haemoglobin type A1c
HDL: High-density lipoprotein
HIS: Hospital information system
IPAQ: International Physical Activity Questionnaires
IT: Information technology
ITT: Intention to treat
LDL: Low-density lipoprotein
MMAS–4: Morisky Medication Adherence Scale—4
NYHA: New York Heart Association
PAT: Provincia Autonoma di Trento
PI: Principal investigator
PPDTA: Percorso Preventivo Diagnostico Terapeutico Assistenziale
QoL: Quality of life
RCT: Randomised controlled trial
sBP: Systolic blood pressure
SD: Standard deviation
SF-12: Short Form—12
SWAT: Study within a trial
SUS: System Usability Scale
T2DM: Type 2 diabetes mellitus
TreC: Cartella Clinica del Cittadino
WHO: World Health Organization
WP: Work package
