# Supplementary material for: Organisational models supported by technology for the management of diabetic disease and its complications in a diabetic clinic setting: study protocol for a randomised controlled trial targeting type 2 diabetes individuals with non-ideal glycaemic values (Telemechron study)
Source: Trials. 2023 Aug 10;24:513. doi: 10.1186/s13063-023-07515-6 (PMC10413726; doi:10.1186/s13063-023-07515-6)
Supplement: Supplementary file 1 — Additional file 1. [file 13063_2023_7515_MOESM1_ESM.pdf]

COMITATO ETICO PER LE SPERIMENTAZIONI CLINICHE

Rep. Int. 9453-30/05/2022
Class. II.12

VERBALE N.5/2022
RIUNIONE DEL 11/05/2022

Il giorno 11 maggio 2022 alle ore 14.00 il Comitato etico per le Sperimentazioni cliniche si riunisce con modalità telematica

(Omissis)

Con la nota di convocazione è stata inviata ai componenti del Comitato etico la documentazione relativa agli argomenti all'ordine del giorno.

Sono presenti alla seduta, quali membri del Comitato etico:

Nome e qualifica	Ruolo secondo D.M. 8 febbraio 2013	Presenza
dott. Giuseppe Moretto, Presidente, neurologo	Esperto clinico (in relazione allo studio di nuove procedure tecniche, diagnostiche e terapeutiche, invasive e semi invasive)	assente
dott. Claudio Graiff, Vicepresidente, oncologo	Clinico	X
prof. Nicola Martinelli, professore di Medicina interna Università di Verona	Clinico	X
prof. Cristiano Chiamulera, professore di Farmacologia Università di Verona	Farmacologo	assente
dott. Carmelo Fanelli, pediatra di libera scelta	Pediatra	assente
dott. Lorenzo Gasperi, Direttore U.O. psichiatria Ambito Est	Clinico	X
dott. Giuliano Mariotti, dirigente medico	Sostituto permanente del direttore sanitario	assente
dott.ssa Giulia Berloffa, Medico di medicina generale	Medico di medicina generale territoriale	X
dott. Enrico Paissan, presidente dell'Associazione Lega Pasi-Battisti	Rappresentante del volontariato o dell'associazionismo di tutela dei pazienti	X
dott.ssa Nicoletta Postal, coordinatore infermieristico ospedale di Arco	Rappresentante dell'area delle professioni sanitarie	X
dott. Riccardo Roni, Direttore Servizio Politiche del farmaco e assistenza farmaceutica	Farmacista del servizio sanitario regionale	X
dott.ssa Chiara Tibaldo, Dirigente farmacista U.O. Farmacia ospedaliera Nord	Esperto in dispositivi medici	X
dott.ssa. Marta Tomasi, docente diritto pubblico, Università di Bolzano	Esperta di bioetica	assente

prof. Giuseppe Verlato, professore di Statistica medica, Università di Verona	Biostatistico	X
dott. Antonio Zuccaro, Funzionario amministrativo	Esperto in materia giuridica e assicurativa	assente

Sono, inoltre, presenti alla seduta, quali membri aggiuntivi del Comitato etico:

Nome e qualifica	Ruolo secondo DM 8 febbraio 2013	Presenza
Ing. Simona Anzivino, Servizio Ingegneria clinica	Ingegnere clinico (in relazione all'area medico-chirurgica oggetto dell'indagine con il dispositivo medico in studio)	assente
dott.ssa Maria Letizia Petroni, Ospedale S.Orsola Malpighi di Bologna	Esperta in nutrizione (in relazione allo studio di prodotti alimentari sull'uomo)	X
dott.ssa Francesca Rivieri, Dirigente medico Servizio clinico di genetica medica	Esperta di genetica (in relazione allo studio di genetica)	X

Segreteria tecnico – scientifica: dott.ssa Francesca Spadaro, farmacista del Servizio Politiche del farmaco e assistenza farmaceutica.
Segreteria amministrativa: dott.ssa Paola Zanetti.

Preso atto della presenza del numero legale dei componenti, alle ore 14.00 la segreteria dichiara aperta la seduta del Comitato Etico.

(Omissis)

I. STUDI SOSPESI/RISPOSTE AL PARERE

(Omissis)

N. registro pareri CE	A765
Codice protocollo	TELEMECHRON
Numero eudraCT	n.a.
Titolo del protocollo	Modelli organizzativi supportati dalla tecnologia per la gestione della malattia diabetica e delle sue complicanze nel setting dell'ambulatorio diabetologico. Uno studio randomizzato controllato su pazienti con diabete mellito tipo 2 affetti da cardiopatia ischemica e/o scompenso cardiaco in controllo glicemico non ideale
Tipo di studio	Non farmacologico sperimentale con utilizzo di App
Fase	n.a.
Promotore	APSS
Centro coordinatore	Dr S.Inchiostro Direttore percorso aziendale di cura del diabete
Sperimentatore principale locale	Marlene Dall'Alda, Massimo Orrasch
Unità Operativa	Geriatria Rovereto, Medicina interna Trento
Responsabile U.O.	Renzo Girardello, Dimitri Peterlana
Numero pazienti previsti nel centro locale	n.d.
Farmaci e materiale forniti dal promotore	-
Prestazioni assistenziali aggiuntive dichiarate	Nessuna
Convenzione economica	n.a.
Costi aggiuntivi dichiarati	Nessuno
Polizza assicurativa	n.a

Nella seduta del 19/01/2022 è stata esaminata la seguente documentazione:

- lettera d'intenti 1/12/2021
- Telemechron protocollo di studio Trento, ver 20211201
- sinossi dello studio
- Telemechron_flowchart
- tabella dati anagrafici Telemechron DIABETE
- tabella dati Telemechron DIABETE
- Tabella tempo 3 mese Telemechron DIABETE
- Telemechron Trento_Informativa e consenso_20211201_ore 15.50
- Questonario SF12 sulla qualità della vita
- Questionario aderenza alla terapia di Morisky-8
- Questionario usabilità –SUS
- Modello _lettera al medico di medicina generale
- Allegato Manuale Paziente Mobile App
- Allegato Manuale operatore sanitario
- Allegato Analisi funzionalità TreC_privacy
- Modello di referto diabetologico
- Duke-Activity-Status-Index (1)
- Del_APSS 440_2020 e allegato
- DGP 1661-2019 progetto telemechron
- Elenco degli allegati
- Moduli 1/Indip (domanda) del 3/21/2021, 2/Indip-ricerca indipendente 1/12/2021, 4-conflitti d'interesse Dall'Alda 1/1272021, 4-conflitti d'interesse Inchiostro 30/11/2021, 4-conflitti d'interesse Orrasch 01/12/2021, 7 (costi aggiuntivi) 1/12/2021

Lo studio è stato presentato dal dott. Sandro Inchiostro.

Di seguito si riporta la valutazione del Comitato etico espressa nella seduta del 19/01/2022:

" Il Comitato etico esprime le seguenti osservazioni:

Aspetti statistici:

Si segnala il seguente refuso presente nella frase: "La significatività statistica delle differenze tra i due gruppi sarà valutata, per le variabili continue con distribuzione normale, mediante test t di Student; in caso di variabili continue con distribuzione non normale, sarà utilizzato il test non parametrico di Wilcoxon dei ranghi con segno". Il test non-parametrico equivalente al test t è il test di Wilcoxon della somma dei ranghi, non il test di Wilcoxon dei ranghi con segno.

Non si replica il calcolo della numerosità campionaria (87 pazienti per gruppo anziché 49).

CALCOLI EFFETTUATI CON IL PROGRAMMA STATA

```
. power twomeans 52 49,sd(7) p(.8)
Estimated sample sizes for a two-sample means test --- t test assuming sd1 = sd2 = sd
Ho: m2 = m1  versus  Ha: m2 != m1
Study parameters:
      alpha =    0.0500
      power =    0.8000
      delta =   -3.0000
         m1 =   52.0000
         m2 =   49.0000
         sd =    7.0000
Estimated sample sizes:   N = 174 ---  N per group = 87
```

Inquadramento normativo e documentazione tecnica:

Il Comitato etico esprime forti perplessità in merito alla dichiarazione che l'App in studio non sia un dispositivo medico. Nel disegno dello studio proposto, l' App non è solo un raccoglitore di dati, ma è anche uno strumento di monitoraggio che fornisce alert.

Ai sensi dell'art 2 del nuovo Regolamento Dispositivi Medici (UE) 2017/745 (MDR) rientra nella definizione di «dispositivo medico» : qualunque strumento, apparecchio, apparecchiatura, software, destinato dal fabbricante a essere impiegato sull'uomo, da solo o in combinazione per la diagnosi, prevenzione, monitoraggio, previsione, prognosi, trattamento o attenuazione di malattie.
Ciò premesso, si invita a presentare la documentazione tecnica in conformità al MDR in quanto la Direttiva 93/42/CEE è abrogata, ed ad avviare la procedura di richiesta di parere al Ministero della Salute.
Coinvolgimento altre U.O./Servizi:
Si invita a coinvolgere il Dipartimento di Tecnologie gestore del sistema TreC/SIO al fine di verificare la compatibilità del sistema di interfaccia in studio App-TreC/SIO e di individuare ed attestare quali moduli sono già istituzionalizzati e gestiti dal Dipartimento e quali sono ancora oggetto di ricerca.
Contapassi:
Si prende atto che per il gruppo di intervento il contapassi è una funzionalità dell'App in studio, mentre per il gruppo di controllo definire quale strumento/applicativo verrà fornito per il conteggio dei passi.
Informativa e consenso:
Si invita a chiarire maggiormente ai partecipanti le modalità di utilizzo del contapassi, soprattutto per il gruppo di controllo e a semplificare, per maggior chiarezza, il modello di consenso informato.
Il Comitato etico, dopo aver valutato gli aspetti etici, metodologici, economici ed assicurativi, esprime
PARERE SOSPENSIVO in attesa del recepimento delle osservazioni sopra riportate"

A seguito del parere sospensivo espresso nella seduta del 19/01/2022 è pervenuta la seguente documentazione:

- Lettera di intenti e sintesi delle correzioni 21.03.2022
- Telemechron protocollo 20220321
- Sinossi dello studio 20220321
- Informativa e consenso 20220321
- tabella dati Telemechron DIABETE 23.02.2022
- questionario ipaq società italiana endocrinologia
- Sintesi incontro Ministero 20220404 final
- Commenti ing. Anzivino

Di seguito si riporta la valutazione (2) del Comitato etico espressa nella seduta del 14/04/2022:
" Il Comitato etico, dopo aver visionato la risposta pervenuta rileva che le indicazioni precedentemente espresse dal comitato in merito all'inquadramento normativo risultano essere esattamente in linea con quanto è stato condiviso col Ministero e che il protocollo risulta ora coerente, mentre permane un'incoerenza nell'informativa per i partecipanti.
Ciò premesso, il Comitato etico, esprime le seguenti osservazioni:
Protocollo:
Per maggior chiarezza si invita ad apportare le seguenti integrazioni
- pag 11: come funzionalità del profilo glicemico si invita a specificare che tale funzione ha l'obiettivo di inserire il profilo glicemico per finalità di mera raccolta e comunicazione, senza generazione di allarmi automatici con conteggi autonomi delle soglie da parte dell'app.
- pag 12: per essere coerenti con le modifiche apportate al protocollo, nell'end-point primario deve essere esplicitato che i valori dell'emoglobina glicata sono quelli ricavati dagli esami/referti ufficiali e non dalle annotazioni riportate nell'app.
Informativa e consenso:
Si rileva che non è stato semplificato, come precedentemente richiesto, il modello di consenso e che in generale, l'informativa appare troppo incentrata nella descrizione delle funzionalità dell'App.
Si invita a rendere coerente con quanto dichiarato nel protocollo gli obiettivi, le funzionalità e la destinazione d'uso dell'app
Dopo aver valutato gli aspetti etici, metodologici, economici ed assicurativi il Comitato etico conferma
PARERE SOSPENSIVO in attesa del recepimento delle osservazioni sopra riportate "

ISO 45001
Sede legale: via Degasperi, 79 - 38123 Trento - P.IVA e C.F. 01429410226 www.apss.tn.it apss@pec.apss.tn.it

A seguito del parere sospensivo espresso nella seduta del 14/04/2022 è pervenuta la seguente documentazione:

- lettera accompagnamento 5.5.2022
- Telemechron protocollo di studio Trento_02.05.2022
- Telemechron Informativa e consenso_04.05.2022

Sono inoltre presenti i commenti dell'Ing. Anzivino

VALUTAZIONE DEL COMITATO ETICO (3)

Viste le correzioni e gli adeguamenti presentati, il Comitato etico esprime

PARERE FAVOREVOLE

Si rammenta che il Comitato Etico deve essere informato dell'inizio della sperimentazione e della sua conclusione o interruzione. Il responsabile della sperimentazione dovrà far pervenire una relazione annuale al Comitato Etico sullo stato della sperimentazione.

(Omissis)

Scritto, letto e confermato,

Il Vice Presidente del Comitato Etico	La verbalizzante
**dott. Claudio Graiff**	**dott.ssa Francesca Spadaro**
Claudio Graiff	Francesca Spadaro

COMITATO ETICO PER LE SPERIMENTAZIONI CLINICHE

Rep. Int. 17489 -11/10/2022
Class. II.12

VERBALE N.8/2022

RIUNIONE DEL 27/09/2022

Il giorno 27/09/2022 alle ore 14.00 il Comitato etico per le Sperimentazioni cliniche si riunisce con modalità telematica.

(Omissis)

Con la nota di convocazione è stata inviata ai componenti del Comitato etico la documentazione relativa agli argomenti all'ordine del giorno. Sono presenti alla seduta, quali membri del Comitato etico:

Nome e qualifica	Ruolo secondo D.M. 8 febbraio 2013	Presenza
dott. Giuseppe Moretto, Presidente, neurologo	Esperto clinico (in relazione allo studio di nuove procedure tecniche, diagnostiche e terapeutiche, invasive e semi invasive)	x
dott. Davide Melisi, oncologo	Clinico	x
prof. Nicola Martinelli, professore di Medicina interna Università di Verona	Clinico	assente
prof. Cristiano Chiamulera, professore di Farmacologia Università di Verona	Farmacologo	assente
dott. Carmelo Fanelli, pediatra di libera scelta	Pediatra	x
dott. Lorenzo Gasperi, Direttore U.O. psichiatria Ambito Est	Clinico	x
dott.ssa Camilla Mattiuzzi, dirigente medico	Sostituta permanente del direttore sanitario	x
dott.ssa Giulia Berloffa, Medico di medicina generale	Medico di medicina generale territoriale	x
dott. Enrico Paissan, presidente dell'Associazione Lega Pasi-Battisti	Rappresentante del volontariato o dell'associazionismo di tutela dei pazienti	assente
dott.ssa Martina Debiasi, infermiere coordinatore col ruolo di tutor clinico presso il Servizio Polo Universitario delle Professioni Sanitarie	Rappresentante dell'area delle professioni sanitarie	x
dott. Riccardo Roni, Direttore Servizio Politiche del farmaco e assistenza farmaceutica	Farmacista del servizio sanitario regionale	x
dott.ssa Chiara Tibaldo, Dirigente farmacista U.O. Farmacia ospedaliera Nord	Esperto in dispositivi medici	x
dott.ssa. Marta Tomasi, docente diritto pubblico, Università di Bolzano	Esperta di bioetica	x
prof. Giuseppe Verlato, professore di Statistica medica,	Biostatistico	x

Università di Verona		
dott. Damiano Luchi, Funzionario amministrativo	Esperto in materia giuridica e assicurativa	assente

Sono, inoltre, presenti alla seduta, quali membri aggiuntivi del Comitato etico:

Nome e qualifica	Ruolo secondo DM 8 febbraio 2013	Presenza
Ing. Simona Anzivino, Servizio Ingegneria clinica	Ingegnere clinico (in relazione all'area medico-chirurgica oggetto dell'indagine con il dispositivo medico in studio)	x
dott.ssa Maria Letizia Petroni, Ospedale S.Orsola Malpighi di Bologna	Esperta in nutrizione (in relazione allo studio di prodotti alimentari sull'uomo)	x
dott.ssa Francesca Rivieri, Dirigente medico Servizio clinico di genetica medica	Esperta di genetica (in relazione allo studio di genetica)	x

Segreteria tecnico – scientifica: dott.ssa Francesca Spadaro, farmacista del Servizio Politiche del farmaco e assistenza farmaceutica.
Segreteria amministrativa: dott.ssa Paola Zanetti.

Preso atto della presenza del numero legale dei componenti, alle ore 14.00 la segreteria dichiara aperta la seduta del Comitato Etico.

(Omissis)

II. EMENDAMENTI SOSTANZIALI (ES)

(Omissis)

N. registro pareri CE - E.S.	ES463
Codice protocollo	**Telemechron**
Numero eudraCT	n.a.
Titolo del protocollo	Modelli organizzativi supportati dalla tecnologia per la gestione della malattia diabetica e delle sue complicanze nel setting dell'ambulatorio diabetologico. Uno studio randomizzato controllato su pazienti con diabete mellito tipo 2 in controllo glicemico non ideale
Autorizzazione studio	Del APSS 440_2020
Tipologia della ricerca	Non farmacologico sperimentale con utilizzo di App
Fase	n.a.
Promotore	APSS
Sperimentatore principale locale	Marlene Dall'Alda, Massimo Orrasch
Unità Operativa	Geriatria Rovereto, Medicina interna Trento
Responsabile U.O.	Renzo Girardello, Cristina Contu
Codice versione e data emend. sostanziale	Prot. Ver 2 09/08/2022
**Sinossi emendamento:** Mod. protocollo v2 09/08/2022 per modifica dei criteri di inclusione. Modifica titolo e tempistiche dello studio .Mod ICF e lettera al MMG	

E' disponibile per la discussione la seguente documentazione:

- lettera Trasmissione 09.08.2022
- protocollo di studio v2_09082022_track changes
- sinossi dello studio v2_09082022_track changes
- tabella dati Telemechron diabete_09082022
- informativa e consenso v2_09082022_track changes
- lettera al medico di medicina generale v2_09.08.2022_track changes
- obiettivi terapeutici in lettera referto v2_09.08.2022_track changes

VALUTAZIONE DEL COMITATO ETICO

Dopo aver visionato le modifiche apportate dall' emendamento in oggetto, il Comitato Etico esprime
PARERE FAVOREVOLE

(Omissis)

Scritto, letto e confermato,

Il Presidente del Comitato Etico	La verbalizzante
**dott. Giuseppe Moretto**	**dott.ssa Francesca Spadaro**
Moretto	Francesca Spadaro

Sede legale: via Degasperi, 79 - 38123 Trento - P.IVA e C.F. 01429410226 www.apss.tn.it apss@pec.apss.tn.it

ETHICS COMMITTEE FOR CLINICAL TRIALS

Rep. Int. 9453-30/05/2022
Class. II.12

Minutes N.5/2022

Meeting 11/05/2022

On the 11/05/2022 at 14.00, the Ethics Committee met online

(Omissis)

With the convocation note, the Ethics Committe members were sent the documents related to the topics on the agenda. Present at the meeting as members of the Ethics Commitee are:

Name and role	Role according to D.M. 8 February 2013	Presence
Dr. Giuseppe Moretto, Chair, neurologist	Clinical expert (in relation to the study of new technical, diagnostic and therapeutic procedures, invasive and semi-invasive)	absent
Dr. Claudio Graiff, Deputy Chair, oncologist	Clinician	X
Prof. Nicola Martinelli, Professor of Internal Medicine, University of Verona	Clinician	X
Prof. Cristiano Chiamulera, Professor of Pharmacology, University of Verona	Pharmacologist	absent
Dr. Carmelo Fanelli, pediatrician	Pediatrician	absent
Dr. Lorenzo Gasperi, Director of the psychiatric operating unit Ambito Est	Clinician	X
Dr. Giuliano Mariotti, Consultant	Permanent substitute of the Medical Director	x
Dr. Giulia Berloffa, general practictioner	General practictioner	X
Dr. Enrico Paissan, Chair of Associazione Lega Pasi-Battisti	Volunteer representative or patient advocacy organisations representative	absent
Dr. Nicoletta Postal, head nurse, Arco hospital	Health professions area representative	X
Dr. Riccardo Roni, Director of the Drug Policy Service and pharmaceutical assistance	Pharmacologist of the regional health service	X
Dr. Chiara Tibaldo, Pharmacist manager of the operating unit Farmacia ospedaliera Nord	Expert in medical devices	X
Dr. Marta Tomasi, public law lecturer Free University of Bolzano	Bioethical expert	X

Prof. Giuseppe Verlato, Professor of statistics in medicine, University of Verona	Biostatistician	X
Dr. Antonio Zuccaro, Administrative officer	Legal and insurance expert	absent

In addition, the following members were invited to participate in the meeting:

Name and role	Role according to DM 8 February 2013	Presence
Engineer Simona Anzivino, Clinical Engineering Service	Clinical engineer (expert in medical device under study)	absent
Dr. Maria Letizia Petroni, Hospital S.Orsola Malpighiof Bologna	Expert in nutrition (in relation to the food study products on humans)	X
Dr. Francesca Rivieri, Clinical Genetic Consultant	Genetic expert (in relation to studies concerned with genetics)	X

Technical-scientific secretariat: Dr. Francesca Spadaro, pharmacist of the Drug Policy Service and pharmaceutical assistance.

Administrative secretariat: Dr. Paola Zanetti.

Noting the presence of a quorum of the members, at 14:00. the secretariat declared open the meeting of the Ethics Committee

(Omissis)

I. PENDING STUDIES/ANSWERS TO OPINION

**Register number EC**	A765
**Protocol ID**	TELEMECHRON
**EudraCT number**	N/A
**Protocol title**	Organisational models supported by technology for the management of diabetic disease and its complications in a diabetic clinic setting. A randomised controlled trial targeting type 2 diabetes individuals and/or heart failure with non-ideal glycemic values
**Type or research**	Non-pharmacological experimental trial with App use
**Phase**	N/A
**Sponsor**	APSS
**Coordinating centre**	Dr. S.Inchiostro, Medical director for the Percorso di cura del diabete
**Local investigators**	Marlene Dall'Alda, Massimo Orrasch
**Operating unit**	Geriatric Unit, Rovereto; Internal Medicine Unit, Trento
**Operating unit directors**	Renzo Girardello, Dimitri Peterlan
**Number of (anticipated) patients by centre**	N/A
**Drugs and materials provided by the sponsor**	None
**Additional care services**	None
**Economic agreement**	N/A
**Additional costs**	None
**Insurance policy**	N/A

In the 19/01/2022 meeting, the following documentation has been evaluated:

- lettera d'intenti 1/12/2021
- Telemechron protocollo di studio Trento, ver 20211201
- sinossi dello studio
- Telemechron_flowchart
- tabella dati anagrafici Telemechron DIABETE
- tabella dati Telemechron DIABETE
- Tabella tempo 3 mese Telemechron DIABETE
- Telemechron Trento_Informativa e consenso_20211201_ore 15.50
- Questonario SF12 sulla qualità della vita
- Questionario aderenza alla terapia di Morisky-8
- Questionario usabilità –SUS
- Modello _lettera al medico di medicina generale
- Allegato Manuale Paziente Mobile App
- Allegato Manuale operatore sanitario
- Allegato Analisi funzionalità TreC_privacy
- Modello di referto diabetologico
- Duke-Activity-Status-Index (1)
- Del_APSS 440_2020 e allegato
- DGP 1661-2019 progetto telemechron
- Elenco degli allegati
- Moduli 1/Indip (domanda) del 3/21/2021, 2/Indip-ricerca indipendente 1/12/2021, 4-conflitti d'interesse Dall'Alda 1/1272021, 4-conflitti d'interesse Inchiostro 30/11/2021, 4-conflitti d'interesse Orrasch 01/12/2021, 7 (costi aggiuntivi) 1/12/2021

The study has been presented by Dr. Sandro Inchiostro.

During the 19/01/2022 meeting, the Ethical Committee expressed the following opinions:

"The Ethics Committee expresses the following observations:

Statistical aspects:

Please note the following typo in the sentence: "The statistical significance of the differences between the two groups will be assessed, for continuous variables with normal distribution, by Student's t test; in the case of continuous variables with non-normal distribution, Wilcoxon's nonparametric test of signed ranks will be used." The nonparametric test equivalent to the t-test is the Wilcoxon test of the sum of ranks, not the Wilcoxon test of ranks with sign

Sample size calculation is not replicated (87 patients per group instead of 49).

Sample size calculation – Stata software

.power twomeans 52 49,sd(7) p(.8)

Estimated sample sizes for a two-sample means test --- t test assuming sd1 = sd2 = sd

Ho: m2 = m1 versus Ha: m2 != m1

Study parameters:

alpha = 0.0500

power = 0.8000

delta = -3.0000

m1 = 52.0000

m2 = 49.0000

sd = 7.0000

Estimated sample sizes: N = 174 --- N per group = 87

Regulatory framework and technical documentation:
The Ethics Committee has strong concerns about the statement that the App under study is not a medical device. In the proposed study, the App appears not only to be a data collection tool, but also a monitoring tool able to send push notifications. According to Article 2 of the new EU Medical Device Regulation 2017/745 a "medical device" is any instrument, apparatus, appliance, software, implant, reagent, material or other, intended by the manufacturer to be used, alone or in combination, for human beings for the diagnosis, prevention, monitoring, prediction, prognosis, treatment or alleviation of disease. That being said, please submit technical documentation in accordance with the MDR 2017/745 as Directive 93/42/EEC has been repealed and initiate the procedure for requesting an opinion from the Ministry of Health.
Other operating units and services involved:
Please involve the Department of Technologies, managing the TreC/HIS system in order to verify the compatibility between the App-TreC interface system and the HIS system and to identify and certify which modules are already institutionalised and handled by the Department, and which are still under study.
Pedometer:
It appears that the pedometer is a feature of the App under study. For the group of control, please define which tool/application will be provided for step counting.
Information note and informed consent form:
More clarification is requested for participants on how to pedometer will be used, in particular for the control group. The informed consent should be simplified for greater clarity.
After ethical, methodological, economical and insurance considerations, the Ethics Committee expresses a ***SUSPENSIVE OPINION pending implementation of the above comments".***

Following the 19/01/2022 statement, the following documentation was provided:

- Lettera di intenti e sintesi delle correzioni 21.03.2022
- Telemechron protocollo 20220321
- Sinossi dello studio 20220321
- Informativa e consenso 20220321
- tabella dati Telemechron DIABETE 23.02.2022
- questionario ipaq società italiana endocrinologia
- Sintesi incontro Ministero 20220404 final
- Commenti ing. Anzivino

The Ethics Committee evaluation (2) from the 14/04/2022 meeting can be found below:
"The Ethics Committee, after reviewing the response received notes that the indications previously expressed by the committee regarding the regulatory framework appear to be exactly in line with what was shared with the Ministry and that the protocol now appears to be consistent, while inconsistency remains in the information note for participants.
That being said, the Ethics Committee expresses the following observations:
Protocol:
For greater clarity, we invite you to add the following explanations:

- *page 11: please specify that the feature regarding the glycemic profile is only meant for mere data collection and communication purposes, without generation of push notifications based on autonomous threshold counts by the App.*
- *page 12: to be consistent with the protocol, it should be made explicit in the primary end-point that the glycated hemoglobin values are those derived from official examinations/referrals and not from the annotations reported in the app.*
- *Information note and informed consent form*

It is noted that the informed consent form and information notice were not simplified as previously requested and that in general, the information notice appears to be to focused on the description of App functionalities. We invite you to align the information with what stated in the protocol (objectives, functionalities and intended use of the App).
After ethical, methodological, economical and insurance considerations, the Ethics Committee confirms a ***SUSPENSIVE OPINION pending implementation of the above comments.***

Following the non-favourable opinion expressed during 14/04/2022 meeting, the following documentation was provided:

- lettera accompagnamento 5.5.2022
- Telemechron protocollo di studio Trento_02.05.2022
- Telemechron Informativa e consenso_04.05.2022

Further comments from Engineer Anzivino are also provided.

ETHICS COMMITTEE EVALUTION (3)

Given the corrections and adaptations submitted, the Ethics Committee expresses a

FAVOURABLE OPINION

We remind you that the Ethics Committee must be informed of the start of the trial and its conclusion or termination. The person in charge of the trial shall send an annual report to the Ethics Committee on the status of the trial

(Omissis)

Written, read and confirmed,

The Ethics Committee Chair deputy	Minutes taker
**Dr. Claudio Graiff**	**Dr. Francesca Spadaro**
[signature]	Francesca Spadaro

ETHICS COMMITTEE FOR CLINICAL TRIALS

Rep. Int. 17489 -11/10/2022
Class. II.12

Minutes N.8/2022

Meeting 27/09/2022

On the 27/09/2022 at 14.00, the Ethics Committee met online.

(Omissis)

With the convocation note, the Ethics Committee members were sent the documents related to the topics on the agenda. Present at the meeting as members of the Ethics Committee are:

Name and role	Role according to D.M. 8 February 2013	Presence
Dr. Giuseppe Moretto, Chair, neurologist	Clinical expert (in relation to the study of new technical, diagnostic and therapeutic procedures, invasive and semi-invasive)	x
Dr. Davide Melisi, oncologist	Clinician	x
Prof. Nicola Martinelli, Professor of Internal Medicine, University of Verona	Clinician	x
Prof. Cristiano Chiamulera, Professor of Pharmacology, University of Verona	Pharmacologist	absent
Dr. Carmelo Fanelli, pediatrician	Pediatrician	x
Dr. Lorenzo Gasperi, Director of the psychiatric operating unit Ambito Est	Clinician	x
Dr. Camilla Mattiuzzi, Consultant	Permanent substitute of the Medical Director	x
Dr. Giulia Berloffa, general practicioner	General practictioner	x
Dr.. Enrico Paissan, Chair of Associazione Lega Pasi-Battisti	Volunteer representative or patient advocacy organisations representative	absent
Dr. Martina Debiasi, Coordinator nurse with the role of clinical tutor at the University of Health Professions (Servizio Polo Universitario delle Professioni Sanitarie)	Health professions area representative	x
Dr. Riccardo Roni, Director of the Drug Policy Service and pharmaceutical assistance	Pharmacologist of the regional health service	x
Dr. Chiara Tibaldo, Pharmacist manager of the operating unit Farmacia ospedaliera Nord	Expert in medical devices	x
Dr. Marta Tomasi, public law lecturer Free University of Bolzano	Bioethical expert	x

Prof. Giuseppe Verlato, Professor of statistics in medicine, University of Verona	Biostatistician	x
Dr. Damiano Luchi, Administrative officer	Legal and insurance expert	absent

In addition, the following members attended the meeting:

Name and role	Role according to DM 8 February 2013	Presence
Engineer. Simona Anzivino, Clinical Engineering Service	Clinical engineer (expert in medical device under study)	x
Dr. Maria Letizia Petroni, Hospital S.Orsola Malpighiof Bologna	Expert in nutrition (in relation to the food study products on humans)	x
Dr. Francesca Rivieri, Clinical Genetic Consultant	Genetic expert (in relation to studies concerned with genetics)	x

Technical-scientific secretariat: Dr. Francesca Spadaro, pharmacist of the Drug Policy Service and pharmaceutical assistance.
Administrative secretariat: Dr. Paola Zanetti.

Noting the presence of a quorum of the members, at 14:00. the secretariat declared open the meeting of the Ethics Committee..

(Omissis)

II. Substantial amendments (SA)

(Omissis)

**Register number EC-SA**	ES463
**Protocol ID**	**Telemechron**
**EudraCT number**	n.a.
**Protocol title**	Organisational models supported by technology for the management of diabetic disease and its complications in a diabetic clinic setting. A randomised controlled trial targeting type 2 diabetes individuals with non-ideal glycemic values
**Study authorisation**	Del APSS 440_2020
**Type of research**	Non-pharmacological experimental trial with App use
**Phase**	n.a.
**Sponsor**	APSS
**Local investigators**	Marlene Dall'Alda, Massimo Orrasch
**Operating unit**	Geriatric Unit Rovereto, Internal Medicine
**Operating unit directors**	Renzo Girardello, Cristina Contu
**Version number and date of substantial amendment**	Prot. Ver 2 09/08/2022
**Reason for admendment request:** Protocol change v2 09/08/2022: change of inclusion criteria. Change of protocol title. Change of Informed Consent Form and GP letter content.	

The following documentation was provided:

- lettera Trasmissione 09.08.2022
- protocollo di studio v2_09082022_track changes
- sinossi dello studio v2_09082022_track changes
- tabella dati Telemechron diabete_09082022
- informativa e consenso v2_09082022_track changes
- lettera al medico di medicina generale v2_09.08.2022_track changes
- obiettivi terapeutici in lettera referto v2_09.08.2022_track changes

ETHICS COMMITTEE EVALUATION

After reviewing the changes requested, the Ethics Committee expresses a **FAVOURABLE OPINION** .

(Omissis)

Written, read and confirmed,

The Ethics Committee Chair

Dr. Giuseppe Moretto

Minutes taker

Dr. Francesca Spadaro
