# Supplementary material for: Organisational models supported by technology for the management of diabetic disease and its complications in a diabetic clinic setting: study protocol for a randomised controlled trial targeting type 2 diabetes individuals with non-ideal glycaemic values (Telemechron study)
Source: Trials. 2023 Aug 10;24:513. doi: 10.1186/s13063-023-07515-6 (PMC10413726; doi:10.1186/s13063-023-07515-6)
Supplement: Supplementary file 2 — Additional file 2. [file 13063_2023_7515_MOESM2_ESM.pdf]

Provincia Autonoma di Trento

AZIENDA PROVINCIALE PER I SERVIZI SANITARI

Trento – via Degasperi 79

VERBALE DI DELIBERAZIONE DEL DIRETTORE GENERALE

Reg. delib. n. 440|2020

OGGETTO: **Approvazione della partecipazione di APSS al Programma di Rete NET-2018-12367206 “Telemedicine for home-based management of patients with chronic diseases and comorbidities: analysis of current models and design of innovative strategies to improve quality of care and optimise resource utilization: TELEMECHRON study” finanziato nell’ambito del “Bando Ricerca Finalizzata 2018” del Ministero della Salute e della relativa convenzione.**
CLASSIFICAZIONE: **1.16**

Il giorno **28/09/2020** nella Sede dell’Azienda Provinciale per i Servizi Sanitari sita in Trento, via Degasperi 79, il dott. Benetollo Pier Paolo, nella sua qualità di

Direttore Generale facente funzioni

ai sensi della deliberazione della Giunta Provinciale di Trento n. 932 del 3 luglio 2020, esamina l’argomento di cui all’oggetto coadiuvato da:

Direttore Sanitario	Direttore Amministrativo	Direttore per l’Integrazione Socio Sanitaria
Dott. Pier Paolo Benetollo	Dott.ssa Rosa Magnoni	Dott. Enrico Nava

OGGETTO: Approvazione della partecipazione di APSS al Programma di Rete NET-2018-12367206 “Telemedicine for home-based management of patients with chronic diseases and comorbidities: analysis of current models and design of innovative strategies to improve quality of care and optimise resource utilization: TELEMECHRON study” finanziato nell’ambito del “Bando Ricerca Finalizzata 2018” del Ministero della Salute e della relativa convenzione.

Il Direttore del Servizio Governance Clinica relaziona quanto segue:

- gli artt. 12 e 12 bis del D. Lgs. 502/1992, come modificato e integrato dal D. Lgs. 299/1999, prevedono il finanziamento a carico del Ministero della Salute di progetti di ricerca finalizzata presentati dai Destinatari Istituzionali, individuati dalla normativa stessa.
- In data 03/04/2018 il Ministero della Salute ha pubblicato il “Bando della Ricerca Finalizzata anno 2018 (esercizi finanziari anni 2016-2017)”, articolato in due principali aree di ricerca, change-promoting e theory-enhancing, ed in cinque categorie di progetti, tra cui i Programmi di Rete (NET).
- Il sopracitato bando prevede che i Programmi di Rete siano articolati in non meno di tre e non più di otto Work Package ed abbiano lo scopo di creare dei gruppi di ricerca e innovazione per lo sviluppo di studi altamente innovativi e caratterizzati dall’elevato impatto sul Servizio Sanitario Nazionale, finalizzati a soddisfare le esigenze di programmazione regionale e di sviluppo dei servizi per il miglioramento dell’assistenza e delle cure offerte; il bando prevede altresì che le proposte di Programmi di Rete afferiscano a specifiche aree tematiche, finanziate dal Ministero della Salute e dalle Regioni interessate a dette tematiche.
- il Ministero della Salute, con decreto direttoriale del 26/07/2019, ha approvato la graduatoria dei progetti ammessi a finanziamento; nell’ambito della tematica n. 5 sopracitata, è risultato collocato nell’area di finanziamento il Programma di Rete NET-2018-12367206 “Telemedicine for home-based management of patients with chronic diseases and comorbidities: analysis of current models and design of innovative strategies to improve quality of care and optimise resource utilization: TELEMECHRON study” (in seguito denominato Programma).
- Il Programma è articolato nei seguenti 4 Work Package (WP):
  - WP 1 (capofila): “Telemedicine for home-based management of patients with chronic kidney diseases and comorbidities: analysis of current models and design of innovative strategies to improve quality of care and optimise resource utilization” - Azienda USL Toscana Nord Ovest - Principal Investigator e Coordinatore del Programma Stefano Bianchi;
  - WP 2: “Assessment of implementation strategies of digital innovations for the continuity of care” – Istituto Superiore di Sanità - Principal Investigator Mauro Grigioni;
  - WP 3: “Telemedicine for home-based management of patients with chronic diseases and comorbidities: analysis of current models, design of innovative strategies for the determinant role of the case manager” - IRCCS Istituti Clinici Scientifici Maugeri SpA - Principal Investigator Simonetta Scalvini;
  - WP 4: “Innovative care models for patients with diabetes to improve the quality of care, empower patients, and optimise resource utilisation” - Azienda Provinciale per i Servizi Sanitari della Provincia Autonoma di Trento - Principal Investigator Silvano Piffer.
- Il finanziamento assegnato, come da Programma approvato, è il seguente:

  a) finanziamento Ministero della Salute: complessivi euro 899.654,00, così suddivisi:

  WP 1: euro 223.666,00;
  WP 2: euro 229.000,00;

WP 3: euro 223.655,00;
WP 4: euro 223.333,00;

b) cofinanziamento Regione Toscana: euro 300.000,00 al WP 1;
c) cofinanziamento Regione Lombardia: euro 300.000,00 al WP 3;
d) cofinanziamento Provincia Autonoma di Trento: euro 299.889,00 al WP 4.

- Ai fini della stipula della convenzione con il Ministero della Salute, come richiesto dal bando, sono state stipulate le convenzioni che regolano i reciproci rapporti inerenti il cofinanziamento regionale.
- Il WP4, coordinato da APSS, prevede il coinvolgimento della Fondazione Bruno Kessler (FBK). APSS è responsabile prevalentemente della componente clinica ed organizzativa dello sviluppo del modello di cura, così come dell'arruolamento e presa in carico dei pazienti tramite il nuovo modello supportato dalla tecnologia. Sarà inoltre coinvolta nella componente di valutazione delle nuove procedure di fornitura di servizi. FBK avrà invece in capo prevalentemente la componente di sviluppo della parte tecnologica del modello di cura (sviluppo dell'App e delle sue funzionalità in particolare) e di analisi dei dati, oltre che alla componente di ricerca in termini di soluzioni anche tecnologiche collegate allo sviluppo degli strumenti informatici utilizzati. Sarà inoltre coinvolta nella componente di valutazione delle nuove procedure di fornitura di servizi.
- È stato perfezionato in data 22/10/2019 il *Consortium Agreement* tra gli Enti sede dei WP in cui si articola il Programma, che regolamenta i rapporti e le modalità di gestione e attuazione del Programma e disciplina i singoli ruoli e compiti nonché gli impegni reciproci; in esso si stabilisce, tra l'altro, che il finanziamento ministeriale sarà trasferito dal Ministero della Salute alla Regione Toscana, la quale provvederà a liquidare direttamente agli Enti sede dei singoli WP le quote spettanti.
- In data 09/07/2020 è stata stipulata la convenzione NET-2018-12367206 tra il Ministero della Salute, la Regione Toscana e, per conoscenza, il Coordinatore del Programma Dott. Stefano Bianchi per regolamentare lo svolgimento del Programma, con particolare riferimento al finanziamento ministeriale.
- Il finanziamento complessivo a favore del WP4 (finanziamento ministeriale e finanziamento provinciale) sarà pari ad euro 523.222,00, dei quali euro 339.733,00 a favore di APSS ed euro 183.489,00 a favore di FBK. La quota di finanziamento Ministeriale, pari ad euro 223.333,00, verrà erogata ad APSS da parte della Regione Toscana. La quota di finanziamento PAT, pari ad euro 299.889,00, verrà invece erogata dalla PAT secondo le seguenti quote: euro 116.400,00 ad APSS ed euro183.489,00 ad FBK.
- Il sopra citato finanziamento dovrà essere utilizzato secondo le modalità definite nelle relative convenzioni e secondo le voci di spesa previste nel piano finanziario di progetto; le suddette spese dovranno essere valutate conformi rispetto alle tipologie acquisite in Azienda da parte del Sevizio aziendale deputato all'acquisto. Tutti gli acquisti dovranno riportare il codice CUP D12F20000870003.
- Il progetto di ricerca prenderà il via in data 01 ottobre 2020 e terminerà in data 30 settembre 2023, salvo eventuali proroghe concesse dalla Ministero della Salute e concordati tra le Parti.

Ciò premesso, è necessario approvare formalmente la partecipazione di APSS al Programma di Rete NET-2018-12367206 "Telemedicine for home-based management of patients with chronic diseases and comorbidities: analysis of current models and design of innovative strategies to improve quality of care and optimise resource utilization: TELEMECHRON study".

È altresì necessario approvare la convenzione con la Regione Toscana che regola le modalità di erogazione del finanziamento ministeriale, di rendicontazione scientifica ed economica, di eventuali modifiche al piano esecutivo e finanziario, nonché di eventuale proroga delle attività e di quanto necessario ai fini della buona conduzione del progetto.

IL DIRETTORE GENERALE FACENTE FUNZIONI

Preso atto della relazione del Direttore del Servizio Governance Clinica;

Fatte proprie le motivazioni espresse dal proponente e condivise dal Direttore competente;

Acquisito il parere favorevole del direttore amministrativo, del direttore sanitario e del direttore per l'integrazione socio sanitaria nella riunione del Consiglio di direzione (verbale rep. n. 47 di data 28/09/2020);

DELIBERA

1. Di approvare la partecipazione di APSS al Programma di Rete NET-2018-12367206 "Telemedicine for home-based management of patients with chronic diseases and comorbidities: analysis of current models and design of innovative strategies to improve quality of care and optimise resource utilization: TELEMECHRON study".
2. Di approvare la convenzione con la Regione Toscana, che allegata alla presente ne costituisce parte integrante e sostanziale, per la disciplina delle attività in capo alle parti e delle modalità di collaborazione.
3. Di dare atto che il Responsabile Scientifico di progetto per APSS il dr. Silvano Piffer, al quale compete garantire le attività progettuali assunte a carico dell'Azienda e le relative attività di rendicontazione nonché, se necessario, sottoporre le attività progettuali alla valutazione preventiva del Comitato Etico per le sperimentazioni cliniche.
4. Di dare atto che per l'esecuzione delle attività progettuali APSS incasserà euro 339.733,00, dei quali euro 116.400,00 da parte della PAT ed euro 223.333,00 da parte della Regione Toscana secondo le modalità stabilite nelle relative Convenzioni.
5. Di assegnare al progetto il codice commessa n. 3 2020 014 (fonte PPA – conto di ricavo 41020170) per la parte di finanziamento PAT e 3 2020 015 (fonte PEN – conto di ricavo 41020070) per la quota di finanziamento della Regione Toscana. Il codice commessa dovrà essere riportato su tutta la documentazione amministrativa di progetto.
6. Di aggiornare il seguente programmi di spesa: 2011/2000 Commesse conto esercizio + 339.733,00 €; non è previsto l'incremento del programma di spesa in c/capitale in quanto non si possono effettuare acquisti in tal senso, come indicato nelle convenzioni di progetto.

Allegato: schema di convenzione.

Inserita da: Dipartimento di Governance / Di Mauro Paola

Verbale letto, approvato e sottoscritto.

Il Direttore Sanitario	Il Direttore Amministrativo	Il Direttore per l’Integrazione socio sanitaria
Benetollo Pier Paolo	Magnoni Rosa	Nava Enrico

Il Direttore Generale facente funzioni
Benetollo Pier Paolo

Il Dirigente del Servizio Affari Generali e Legali
Toniolatti Armando

Autonomous Province of Trento

AZIENDA PROVINCIALE PER I SERVIZI SANITARI

Trento – via Degasperi 79

MINUTES OF THE GENERAL MANAGER’S DELIBERATION

Reg. delib. n. 440|2020

SUBJECT: **Approval of the participation of APSS in the NET-2018- 12367206 Network Program “Telemedicine for home-based management of patients with chronic diseases and comorbidities: analysis of current models and design of innovative strategies to improve quality of care and optimise resource utilization: TELEMECHRON study” funded under the “*Bando Ricerca Finalizzata 2018*” of the Ministry of Health and related agreement.**
CLASSIFICATION: **1.16**

On **28/09/2020** at Sede of Azienda Provinciale per i Servizi Sanitari located in Trento, via Degasperi 79, Dr. Benetollo Pier Paolo, in his position of

Acting General Manager

In accordance with the resolution of the Giunta Provinciale di Trento No. 932 of July, 3, 2020, examines the subject matter of the meeting supported by:

Health Director	Administrative Director	Director for Social and Health Integration
Dr. Pier Paolo Benetollo	Dr. Rosa Magnoni	Dr. Enrico Nava

SUBJECT: **Approval of the participation of APSS in the NET-2018- 12367206 Network Program “Telemedicine for home-based management of patients with chronic diseases and comorbidities: analysis of current models and design of innovative strategies to improve quality of care and optimise resource utilization: TELEMECHRON study” funded under the “*Bando Ricerca Finalizzata 2018*” of the Ministry of Health and the related agreement.**

The Director of the Clinical Governance Service reports the following:

- Articles 12 e 12 bis of Legislative Decree 502/1992, as amended and complemented by Legislative Decree 299/1999, establish the financing at the expense of the Ministry of Health of finalized research projects submitted by Institutional Recipients, identified by the same legislation.
- On 03/04/2018, the Ministry of Health published the "Notice of Finalized Research Year 2018 (fiscal years 2016-2017)," divided into two main areas of research, change-promoting and theory-enhancing, and five categories of projects, including Network Programs – *Programmi di Rete (NET).*
- The above-mentioned call establishes that the Network Programs are divided into no less than three and no more than eight Work Packages and are aimed at creating research and innovation groups for the development of highly innovative studies characterized by their high impact on the National Health Service, aimed at meeting the needs of regional planning and service development for the improvement of the care and treatment offered; the call also establishes that the Network Program proposals pertain to specific thematic areas, funded by the Ministry of Health and the Regions interested in those topics.
- The Ministry of Health, by Directorial Decree 26/07/2019, approved the list of projects accepted for funding; under the above-mentioned topic No.5, the NET-2018-12367206 Network Program “Telemedicine for home-based management of patients with chronic diseases and comorbidities: analysis of current models and design of innovative strategies to improve quality of care and optimise resource utilization: TELEMECHRON study” (hereinafter referred to as Program) has been included in the funding agreement.
- The Program is structured into the following 4 Work Packages (WPs):
  - WP 1 (coordinator): “Telemedicine for home-based management of patients with chronic kidney diseases and comorbidities: analysis of current models and design of innovative strategies to improve quality of care and optimise resource utilization” - Azienda USL Toscana Nord Ovest - Principal Investigator and Program coordinator Stefano Bianchi;
  - WP 2: “Assessment of implementation strategies of digital innovations for the continuity of care” – Istituto Superiore di Sanità - Principal Investigator Mauro Grigioni;
  - WP 3: “Telemedicine for home-based management of patients with chronic diseases and comorbidities: analysis of current models, design of innovative strategies for the determinant role of the case manager” - IRCCS Istituti Clinici Scientifici Maugeri SpA - Principal Investigator Simonetta Scalvini;
  - WP 4: “Innovative care models for patients with diabetes to improve the quality of care, empower patients, and optimise resource utilisation” - Azienda Provinciale per i Servizi Sanitari della Provincia Autonoma di Trento - Principal Investigator Silvano Piffer.
- The funding allocated, as per the approved Program, is as follows:

  a) Ministry of Health funding: a total of 899.654,00 euros, allocated as follows:

  WP 1: 223.666,00 euros;

  WP 2: 229.000,00 euros;

WP 3: 223.655,00 euros;
WP 4: 223.333,00 euros;

b) co-financing Region of Tuscany: 300.000,00 euros to WP 1;
c) co-financing Region of Lombardy: 300.000,00 euros to WP 3;
d) co-financing Autonomous Province of Trento: 299.889,00 euros to WP 4.

- For the purpose of the agreement with the Ministry of Health, as required by the call, the agreements regulating the mutual relations pertaining to regional co-financing were stipulated.
- WP4, coordinated by APSS, establishes the involvement of the Bruno Kessler Foundation (FBK). APSS is primarily responsible for the clinical and organizational component of the development of the care model, as well as the enrollment and care of patients through the new technology-supported model. It will also be involved in the evaluation component of the new service delivery procedures. On the other hand, FBK will primarily be in charge of the development component of the technological part of the care model (development of the App and its features in particular) and data analysis, as well as the research component in terms of solutions including technological solutions related to the development of the IT tools used. It will also be involved in the evaluation component of new service delivery procedures.
- A Consortium Agreement was finalised on 22/10/2019 between the bodies of the WPs into which the Program is subdivided, regulating the relationships and modalities of management and implementation of the Program and governing individual roles and tasks as well as mutual commitments; it stipulates, among other things, that ministerial funding will be transferred from the Ministry of Health to the Region of Tuscany, which will directly transfer to the bodies of the individual WPs the amounts due.
- On 09/07/2020, the agreement NET-2018-12367206 was signed between the Ministry of Health, the Region of Tuscany and, for information, the Program Coordinator Dr. Stefano Bianchi to regulate the conduct of the Program, with particular reference to ministerial funding.
- The total funding in favour of WP4 (Ministerial funding and provincial funding) will be euro 523,222.00, of which euro 339,733.00 in favour of APSS and euro 183,489.00 in favour of FBK. The Ministerial funding share, amounting to euro 223,333.00, will be granted to APSS by the Tuscany Region. The PAT funding share of euro 299.889,00 will be granted by PAT according to the following shares: euro 116.400,00 to APSS and euro 183.489,00 to FBK.
- The aforementioned funding shall be used in accordance with the terms defined in the relevant agreements and according to the items of expenditure stipulated in the project financial plan; the aforementioned expenditures shall comply with the types acquired in the *Azienda* by the *Servizio aziendale* in charge of purchases. All purchases should bear the CUP D12F20000870003.
- The research project will start on October 01, 2020 and end on September 30, 2023, unless extended by the Ministry of Health and agreed upon by the Parties.

That being said, it is necessary to formally approve the participation of APSS in the NET-2018-12367206 Network Program “Telemedicine for home-based management of patients with chronic diseases and comorbidities: analysis of current models and design of innovative strategies to improve quality of care and optimise resource utilization: TELEMECHRON study”.

It is also necessary to approve the agreement with the Region of Tuscany that regulates how the ministerial funding will be disbursed, scientific and economic reporting, any changes to the executive and financial plan, as well as any extension of activities and all that is necessary for the proper conduct of the project.

ACTING GENERAL DIRECTOR

Having considered the report of the Director of the Clinical Governance Service;

Having endorsed the reasons expressed by the proposer and by the Director responsible;

Having acquired the favourable approval of the administrative director, the medical director and the director for social and health integration during the meeting of *Consiglio di direzione* (minutes rep. No. 47 dated 28/09/2020);

DECIDES

1. To approve the participation of APSS in the NET-2018-12367206 Network Program “Telemedicine for home-based management of patients with chronic diseases and comorbidities: analysis of current models and design of innovative strategies to improve quality of care and optimise resource utilization: TELEMECHRON study”.
2. To approve the agreement with the Region of Tuscany, which attached to this document is an integral and substantive part, for the regulation of the activities in charge of the parties and the modalities of collaboration.
3. To acknowledge Dr. Silvano Piffer as the Scientific Project Manager for APSS, who is responsible for ensuring the project activities for the Azienda and the related reporting activities as well as, if necessary, submitting the project activities for prior evaluation by the Ethics Committee for Clinical Trials.
4. To acknowledge that for the execution of the project activities APSS will receive 339,733.00 euros, of which 116,400.00 euros from the PAT and 223,333.00 euros from the Region of Tuscany in accordance with the modalities established in the relevant Agreements.
5. To assign the Project Code No. 3 2020 014 (source PPA – revenue account 41020170) to the project for the PAT funding portion and No. 3 2020 015 (source PEN – revenue account 41020070) for the Tuscany Region funding portion. The Project Code should be reported on all project administrative documentation.
6. To update the following expenditure program: 2011/2000 Order Code income + 339.733,00 €; no increase in the capital spending program is foreseen, as indicated in the project agreements.

Attached: outline of agreement

Submitted by: Department of Governance / Di Mauro Paola

Minutes read, approved and signed.

Health Director	Administrative Director	Director for Social and Health Integration
Benetollo Pier Paolo	Magnoni Rosa	Nava Enrico

Acting General Manager
Benetollo Pier Paolo

The Director of the General and Legal Affairs Service
Toniolatti Armando
